# Evidence that cholinergic mechanisms contribute to hyperexcitability at early stages in Alzheimer’s disease

**DOI:** 10.3389/frdem.2025.1513144

**Published:** 2025-06-17

**Authors:** Helen E. Scharfman, Korey Kam, Áine M. Duffy, John J. LaFrancois, Paige Leary, Elissavet Chartampila, Stephen D. Ginsberg, Christos Panagiotis Lisgaras

**Affiliations:** ^1^Center for Dementia Research, The Nathan Kline Institute of Psychiatric Research, Orangeburg, NY, United States; ^2^Department of Child and Adolescent Psychiatry, New York University Grossman School of Medicine, New York, NY, United States; ^3^Department of Psychiatry, New York University Grossman School of Medicine, New York, NY, United States; ^4^Department of Neuroscience and Physiology, New York University Grossman School of Medicine, New York, NY, United States; ^5^The NYU Neuroscience Institute, New York University Grossman School of Medicine, New York, NY, United States

**Keywords:** cholinergic, muscarinic, memory, amyloid β, mouse model, Tg2576, down syndrome, Ts65Dn

## Abstract

A long-standing theory for Alzheimer’s disease (AD) has been that deterioration of synapses and depressed neuronal activity is a major contributing factor. We review the increasing evidence, in humans and in mouse models, that show that there is often neuronal hyperactivity at early stages rather than decreased activity. We discuss studies in mouse models showing that hyperexcitability can occur long before plaque deposition and memory impairment. In mouse models, a generator of the hyperactivity appears to be the dentate gyrus. We present evidence, based on mouse models, that inhibition of muscarinic cholinergic receptors or medial septal cholinergic neurons can prevent hyperactivity. Therefore, we hypothesize the novel idea that cholinergic neurons are overly active early in the disease, not depressed. In particular we suggest the medial septal cholinergic neurons are overly active and contribute to hyperexcitability. We further hypothesize that the high activity of cholinergic neurons at early ages ultimately leads to their decline in function later in the disease. We review the effects of a prenatal diet that increases choline, the precursor to acetylcholine and modulator of many other functions. In mouse models of AD, maternal choline supplementation (MCS) reduces medial septal cholinergic pathology, amyloid accumulation and hyperexcitability, especially in the dentate gyrus, and improves cognition.

## Introduction

Here we focus on early stages of AD, a time before amyloid plaques and neurofibrillary tangles have developed. The reason for this emphasis is that understanding the early stages might allow a greater opportunity to develop treatments to prevent or delay AD. Indeed, it has already been suggested that treating AD early is likely to be more effective than starting a therapeutic strategy at late stages (Guest et al., 2020; Zhou et al., 2022).

## Hyperactivity in AD

One of the areas of AD research that has captured increasing attention is the idea that hyperactivity is a characteristic of AD. Note that for the present purposes, the term “hyperactivity”” is used interchangeably with “hyperexcitability.” Both hyperactivity and hyperexcitability are commonly defined as neuronal activity that is greater than the normal range, like the aberrant neuronal activity observed in epilepsy. One type of abnormal activity includes large and short-lasting spikes in the electroencephalography (EEG) which occur between seizures, called interictal spikes (IIS). They may also be called interictal epileptiform discharges (IEDs), especially if they are more complex electrophysiologically than a single spike. In the work of our laboratory described below we mainly use the term IIS because typically the waveforms are not complex. Importantly, some spikes in the EEG are normal, so to distinguish those that are abnormal we used multiple electrodes and define an IIS as occurring in all electrodes synchronously. Both IIS and IEDs are much shorter than the most well-known type of hyperexcitability in epilepsy, which is a seizure.

It has been known for decades that some individuals with AD have IIS, IEDs, and seizures (Gimenez-Roldan et al., 1971; Hauser et al., 1986; Risse et al., 1990; Romanelli et al., 1990; Mendez and Lim, 2003). These observations were not considered relevant to AD when they were first observed because seizures are common in the normal elderly population (Hauser and Kurland, 1975; Hauser, 1978), so seizures were not considered to reflect AD necessarily. Furthermore, initial studies of seizures in AD found the seizures in individuals with a familial form of AD (Bird et al., 1989; Kennedy et al., 1993; Rainero et al., 1994; Mangone et al., 1995; Ezquerra et al., 1999) which is a small subset of AD. In contrast, most individuals have sporadic AD. However, more recordings of the EEG in individuals with AD have now revealed that IIS, IEDs and seizures may occur not only in familial but also sporadic AD. In a recent study of patients with AD and no history of risk factors for epilepsy, epileptiform activity in the EEG was present in 22% (Lam et al., 2020). In other studies, the frequency was as high as 54% (Vossel et al., 2016).

These numbers may be an underestimation because hyperexcitability in AD is often missed. Reasons for this have been discussed (Palop and Mucke, 2009; Lam et al., 2020). One reason is that IIS and IEDs occur without any visible sign, and seizures can be non-convulsive or occur in sleep. In most individuals, EEG is not conducted in patients with AD. Even if a scalp EEG is performed, it may only be conducted for a short time. Moreover, some abnormal activity in AD is deep in the brain, hard to detect from the scalp (Lam et al., 2017).

Hyperactivity in AD is potentially an important part of the disease because it appears to hasten disease progression. Thus, patients had a faster decline after an initial seizure than patients without epileptiform activity (Volicer et al., 1995). A comparison of AD patients with and without evidence of epilepsy showed those with epilepsy had an earlier cognitive decline (Vossel et al., 2013). These studies suggested that seizures were not an epiphenomenon, as previously assumed, but contributed to disease progression. One reason seizures may worsen symptoms in AD is based on studies in rodents. When seizures were induced experimentally in normal rodents, there was an increase in the release of amyloid β (Aβ) into the extracellular space (Kamenetz et al., 2003; Cirrito et al., 2005). In a mouse model of AD, increasing neuronal activity had a similar effect (Bero et al., 2011). Seizures may also worsen symptoms in AD because they change gene expression throughout the brain, activate microglia, and trigger inflammation (Brooks-Kayal et al., 2009; Wilcox and Vezzani, 2014; Broer and Pauletti, 2024), all of which have been implicated in AD.

IIS also have been shown to impair memory in rodents and in humans (Kleen et al., 2010; Kleen et al., 2012; Kleen et al., 2013) so they may also contribute to the progressive memory impairment in AD. The experiments that used rats were conducted as they were developing experimental epilepsy, having already had a brain insult to initiate epileptogenesis (Kleen et al., 2010). In humans, patients with epilepsy were tested between seizures. IIS disrupted cognitive tasks such as retrieval (Kleen et al., 2012, 2013). IIS, IEDs and seizures may also contribute to progressive pathology in AD by disrupting sleep, a time when they are common, and also a time when Aβ clearance occurs (Nedergaard and Goldman, 2020). Furthermore, EEG abnormalities in sleep could disrupt the process of memory consolidation at that time.

One of the advances that led to more attention to hyperactivity in AD was a study showing that in the early prodromal stage of AD called mild cognitive impairment (MCI), amnestic individuals showed hyperactivity in fMRI. Importantly, these individuals were administered an anticonvulsant, which reduced hyperactivity, and also improved cognition (Bakker et al., 2012). The anticonvulsant was levetiracetam, which is notable because other studies have shown that patients with AD that exhibit epileptiform activity benefit from levetiracetam treatment, and levetiracetam did not show a detectable benefit when tested in those patients without evidence of epileptiform activity (Vossel et al., 2021).

Improvements in the ability to study mouse EEG has been important to bring more attention to the seizures in AD. The advent of EEG recording in mice made it clear how common seizure activity was in the mouse models of AD. Importantly, seizures were not always associated with convulsions, reinforcing the idea that in humans there may be an underestimation of seizures. Most of the initial studies with EEG were conducted in mice with overexpression and mutation in human amyloid precursor protein (hAPP) which promotes the amyloidogenic processing of APP and increase amounts of Aβ as animals age (Palop et al., 2007). However, hyperexcitability has also been found in models of tauopathies. In a mouse that simulates frontotemporal dementia (FTD) with parkinsonism-linked to chromosome 17 (FTDP-17), where patients have seizures (Sperfeld et al., 1999), mice show epileptiform activity and seizures also (Sanchez et al., 2018). Mice with a P301S mutation in the gene encoding tau (microtubule-associated protein tau; MAPT) that is associated with FTD and corticobasal degeneration (CBD) shows seizures (Bugiani, 2000). The rTg4510 mouse model expresses tau with a P301L mutation and shows increased susceptibility to kindled seizures (Liu et al., 2017), increased glutamate release (Taipala et al., 2022) and increased neuronal excitability *in vitro* (Hole et al., 2024), consistent with single cell electrophysiology showing increased cortical excitability (Luebke et al., 2010). Tau is very interesting because tau deletion in hAPP mice can protect against hyperexcitability (Roberson et al., 2007; Roth et al., 2024) and selective deletion of tau in a mouse model of Dravet syndrome protects against seizures (Shao et al., 2022). However, tau is also complicated because in the same mouse model which showed greater excitability, the rTg4510 mouse, young mice showed reduced excitability (Xolalpa-Cueva et al., 2022). The variability in the published studies, some showing reduced and others increased excitability, could be due to different brain locations that were studied. For example, hyperactivity may occur in cortex but not hippocampus, or vice-versa. Also there could be decreased excitability at an early age and higher excitability later, or vice versa. Finally, extrapolating these studies to our understanding of how neurofibrillary tangles affect the brain is difficult, although one can learn a great deal about tau and hyperphosphorylated tau in the absence of Aβ accumulation.

Mice with multiple mutations, to produce increased levels of Aβ and hyperphosphorylated tau, provide opportunities to study interactions that are absent in mice with only an increase in Aβ or only an increase in pathological forms of tau. Such interactions are important because both occur in the disease. The mice with multiple mutations usually have at least one hAPP mutation, mutation in tau, and a mutation in presenilin 1 (3 mutations, 3xTg; 5 mutations, 5xTg). Mice which have risk factors for AD, such as the APOE4 allele, also are relevant because these mice exhibit increased excitability (Hunter et al., 2012; Paesler et al., 2015; Siwek et al., 2015; Lamoureux et al., 2021; Vande Vyver et al., 2022; Barbour et al., 2024), suggesting that there are many reasons for epileptiform activity in AD.

It is important to consider that none of the mouse models are ideal simulations of AD. One criticism has been that overexpression of mutant APP typically uses a promoter other than the APP promoter, which can lead to artifacts. Therefore, mice were developed that are “knockins” of mutant APP and use the APP promoter (Nilsson et al., 2014; Saito et al., 2014). One of the most common knockin models is the *App*^NL-G-F^ where there are 3 familial mutations (NL, Swedish; G, Artic; F, Iberian) in hAPP. Initially these mice did not show epileptiform activity, which suggested that studies of hyperexcitability in mice reflected an artifact (Brown et al., 2018). However, additional studies of the mice showed hyperexcitability in the form of epileptiform spikes, with frequencies comparable to mouse models with overexpression of hAPP using a promoter other than the APP promoter. One such mouse is the J20 (hAPP with the Swedish and Indiana mutations, driven by the PDGFb promoter) or I5 (wild-type hAPP driven by the PDGFb promoter (J20, I5; Johnson et al., 2020). Therefore, hyperexcitability does not seem to be an artifact of the hAPP models that use a promoter other than the APP promoter.

Another criticism of mouse models is that hyperexcitability in mouse models appears to be mainly in rapid eye movement sleep (REM) whereas in AD the epileptiform activity occurs primarily in slow-wave sleep (non-REM; NREM; Brown et al., 2018). However, at least in the Tg2576 mouse, IIS occur in both REM and non-REM sleep (Kam et al., 2016). At early ages IIS are mostly in REM, but later in life, IIS in NREM are increasingly common (Kam et al., 2016). This is interesting because patients are primarily assessed after diagnosis, not at the very early stages analogous to what we have studied in Tg2576 mice. If humans could be studied at early ages, prior to memory impairment and detectable Aβ accumulation, epileptiform activity might be detected primarily in REM, like the mice.

## Understanding initial stages of hyperactivity in AD

To understand the relationship of hyperactivity to AD better we focused on early stages so there would be fewer of the complex pathological changes that occur at older ages. We wanted to know at what age we would first detect hyperexcitability whether it would be manifested as IIS, IEDs, or seizures. If we found hyperexcitability at early ages the results would be important because they would argue against the idea that hyperactivity was simply a consequence of other pathology. We thought a mouse study would help address the issue because we could implant electrodes for EEG at very young ages before memory loss and plaques are evident.

For these experiments we used the Tg2576 mouse because it develops neuropathology slowly. The slow nature of the progression would allow us to test the hypothesis that hyperexcitability would occur before amyloid deposition as plaque. The Tg2576 mouse overexpresses APP695, the primary type of APP in the brain and in neurons (Nalivaeva et al., 2012), with a form of APP called APPSwe because it was found in a Swedish cohort with AD (Hsiao et al., 1996). In the mouse model, mutant APP is expressed by the hamster prion promoter so it occurs widely in the brain (Hsiao et al., 1996). There are two mutations in APP (KM670/671NL) that facilitate metabolism to Aβ1-42, the form of Aβ commonly considered to be most harmful. Aβ plaques begin by approximately 6 months of age and are robust after 12 months (Hsiao et al., 1996; Irizarry et al., 1997; Alcantara-Gonzalez et al., 2021; Criscuolo et al., 2024).

We began by examining mice at an age we thought would show no abnormal excitability—approximately 1 month of age or > 6 months before amyloid plaques can be detected in Tg2576 mice. The 1 month age is also a time when memory impairment in mouse models of AD has not been reported. In Tg2576 mice, the first impairments have been found at 3–4 months of age, using tasks that probe novel object location memory (Duffy et al., 2015). Another study found defects in contextual fear conditioning at 5 months of age (Jacobsen et al., 2006). Most investigators suggest memory is first affected much later, after 6 months, but these studies often use other types of cognitive tasks or simply do not test at early ages, assuming cognitive impairment occurs later.

Electrodes were implanted to sample areas of the brain implicated in AD, including hippocampus and cortex. Therefore, 4 electrodes were used: a subdural electrode was placed over the frontal cortex, another over the occipital cortex, and one depth electrode was placed in each dorsal hippocampus. Animals were allowed to recover for 1 week before recording. We also used mice that had subdural electrodes over hippocampus instead of depth electrodes because we did not know if trauma to the hippocampus would influence the recordings. Fortuitously it did not, so data from mice with subdural and depth electrodes were pooled. Nevertheless, we also used a non-invasive method to study neuronal activity, immunocytochemistry using c-Fos and ΔFosB as markers of acute and chronic neuronal activity (respectively; Herrera and Robertson, 1996; McClung et al., 2004).

We recorded for 24 h continuously at 5 weeks of age, and then repeated the 24 h recording at 2, 3, 4, 5, and 6 months of age. We also added older ages, up to 24 months. Twenty-four hours was chosen so that we could sample the major behavioral states: exploration, immobility and sleep. The EEG was used to assess behavioral state, with a high theta/delta ratio to define exploration and REM sleep and high delta activity for REM NREM sleep. To discriminate sleep from wakefulness we used a light-emitting diode (LED) implanted between the shoulder blades to serve as a motion detector.

**Figure 1:**
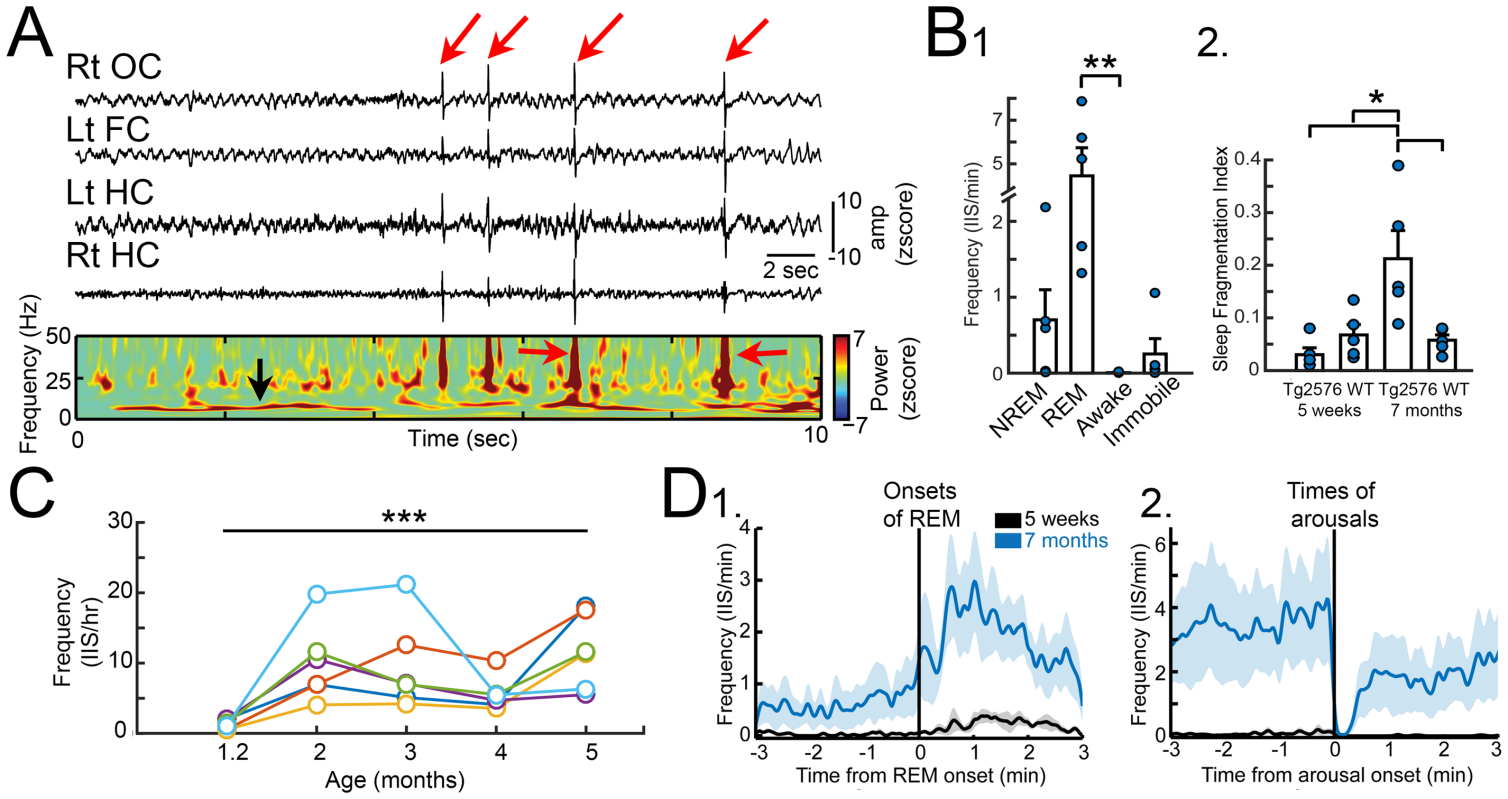
**(A)** A recording from a Tg2576 mouse using a subdural screw over the right occipital cortex (Rt OC), left frontal cortex (Lt FC), and two depth electrodes in each dorsal hippocampus (Lt HC, Rt HC). Large brief deflections are IIS (red arrows). Below the recordings is a spectrogram showing the increased power during the IIS (red arrows). The power at ~8 Hz (black arrow) reflects high power in the theta band, which characterizes REM sleep. **(B)** 1. Quantification of IIS in each behavioral state at 5 weeks of age in Tg2576 mice (n = 5) shows significantly higher IIS rates in REM compared to wakefulness. 2. The sleep fragmentation index showed no significant differences between Tg256 and WT mice at 5 weeks of age but there was a higher index at 7 months of age. **(C)** IIS rates for 5 mice (each mouse is shown by a different color). Rates were calculated from a 24 h period at 5 weeks and then the same mouse was recorded at 2, 3, 4, and 5 months of age. The results show a rise in rates from 1–3 months but after 3 months the rates are variable and tend to decline. **(D)** 1. IIS rates are plotted for 5 week (black) and 7 months-old (blue) Tg2576 mice showing that as REM began there was an increase in IIS rates. 2. At the time of arousal, IIS rates declined sharply. Graphs show mean ± SEM. Adapted from Kam et al. (2016).

Remarkably, the earliest recordings, at 5 weeks of age, showed abnormal EEG in the Tg2576 mice (Figure 1A; Kam et al., 2016). To date we have not detected abnormal events in controls. At 5 weeks of age, the most common abnormality was a large spike that occurred almost synchronously across all 4 electrodes, and they occurred primarily in REM sleep (Figure 1B). Importantly, another group showed independently that spikes occurred spontaneously in the EEG of 6 weeks-old Tg2576 mice (Bezzina et al., 2015).

We found the numbers of large spikes were low at 1 month of age, but they increased with age (Figure 1C) and persisted at variable rates for the lifespan. IIS also began to develop more in NREM sleep with age. In contrast, exploration was a time when IIS were absent. In contrast, none of the WT mice showed aberrant activity at any time or any age, even >15 months (Kam et al., 2016).

Interestingly, seizures were rare relative to IIS. When we recorded continuously for 2 weeks in the 1–2 months-old mice or 6 months-old mice, there were no seizures (Kam et al., 2016; Chartampila et al., 2024). Nevertheless, we observed convulsive seizures in other mice that were not recorded, although rarely. These seizures often were severe and ended in death. At the time of death, mice were in a posture that reflects death during a seizure, a position with extended limbs. When we examined the mouse, there was a postmortem delay that prevented extensive study of the brain. In the periphery, major organs did not appear to be affected. Other investigators have also found that premature, sudden death occurs in another mouse model (Huang and Lemke, 2022). These mice could be models of sudden unexplained death in epilepsy (SUDEP) which is a serious clinical concern (Duncan and Brodie, 2011). Sudden death also occurs in AD (Opeskin, 1996) but less is known about it.

Other animal models of AD also show seizures, although few studies have conducted video-EEG for long enough to define the frequency of seizures. In one study that did, the majority of mice had few seizures (Minkeviciene et al., 2009). This was a study of a hAPP mouse with the Swedish mutation and a presenilin 1 mutation (APP/PS1). Together, the data suggest that IIS are more common than seizures in mouse models. That is interesting if it also occurs in human AD, because it would differentiate AD from epilepsy. In other words, although IIS and seizures occur in both diseases, seizures may define epilepsy whereas epileptiform activity may define AD.

Interestingly, it has been suggested that IIS disrupt memory retrieval while awake, as discussed above (Kleen et al., 2010). Furthermore, REM sleep appears to be important to memory consolidation (Almeida-Filho et al., 2018; Scarpelli et al., 2019; Mizuseki and Miyawaki, 2023). Therefore, IIS might disrupt memory in AD by effects during wakefulness and by interfering with memory consolidation during sleep. IIS may also disrupt clearance of Aβ during sleep because it has been suggested that Aβ is cleared from the brain during this time (Nedergaard and Goldman, 2020).

Indeed, by 7 months of age the Tg2576 mice show sleep fragmentation, although not at 5 weeks (Figure 1B; Kam et al., 2016). Therefore, IIS at 1 month of age are not caused by disrupted sleep and are not able to disrupt sleep yet. However, IIS may influence sleep (and vice-versa) at a later age. This is important because it may explain why there is variability in IIS numbers at ages after 4 months. For example, if there is less sleep there might be less time for IIS to occur, making IIS numbers appear to drop.

## Additional mouse models

We examined several other mouse models besides Tg2576 mice and found similar results. Therefore we do not think the results are model-specific. One mouse model we examined had the same hAPP mutation, the Swedish mutation, but the promoter was the mouse Thy1 promoter. This mouse line is commonly called APP23. The background of the APP23 mice is C57BL6/J whereas the Tg2576 background is SBJL. APP23 mice were remarkably similar to the Tg2576 mouse, showing IIS primarily in REM sleep at just 5 weeks of age (Kam et al., 2016). Therefore, the promoter that overexpressed the transgene, and the background, did not seem to play a major role in IIS.

We also used a mouse that overexpressed human WT APP (isoform 751) using the Thy1.2 promoter. In 5 mice, 3 developed IIS and the earliest time that was studied was 5 months of age (Kam et al., 2016). IIS were also detected in the I5 line, which overexpresses human WT APP by the platelet derived growth factor (PDGF) promoter (Mucke et al., 2000). They develop high levels of APP but they do not develop plaques. These studies are important because they argue against a role of APP mutation in causing hyperexcitability. They suggest instead the intriguing possibility that accumulation of APP may be sufficient for hyperexcitability. Indeed, high levels of APP could have many effects on excitability directly, or by its metabolites (Mattson, 1997).

The possibility that APP overexpression could lead to hyperexcitability made us interested in mouse models of Down syndrome. Down syndrome is relevant to AD, because almost all individuals with Down syndrome develop AD (Price et al., 1982; Head et al., 2012; Salehi et al., 2016). AD in Down syndrome is often attributed to elevation of APP because the APP gene is on human chromosome 21 which is triplicated in Down syndrome. However, several other genes on chromosome 21 are triplicated and have been suggested to play a role in Down syndrome (Liu et al., 2022). There are many mouse models of Down syndrome and the one we selected was the Ts65Dn mouse. This mouse is trisomic for segments of mouse chromosome 16 and 17 (MMU16 and 17), which are orthologs of chromosome 21 in humans (Davisson et al., 1990). The mice recapitulate several of the characteristics of human Down syndrome such as cognitive impairment and cholinergic neuronal loss with age (Galdzicki and Siarey, 2003; Alldred et al., 2015a, 2015b). However, the Ts65Dn mouse model has been criticized because the mice do not simulate triplication of chromosome 21 exactly (Herault et al., 2017; Farrell et al., 2022). We found that Ts65Dn mice develop IIS and they are primarily in REM, like the Tg2576 mouse. IIS were found at early ages and throughout life (Lisgaras and Scharfman, 2023b).

In epilepsy there are multiple mechanisms that can cause IIS, IEDs, and seizures (Staba and Bragin, 2011; Levesque et al., 2019; Lai et al., 2023; Levesque et al., 2023; Prince and Avoli, 2024). Therefore we were also interested in studying a mouse model without detectable APP and Aβ elevation. One mouse model that interested us was a mouse line without presenilin 2 (PS2), because it showed increased seizure susceptibility (Bellio et al., 2024). There are two presenilins, PS1 and PS2, and both are part of the γ secretase complex involved in APP metabolism. Notably, γ secretase does not only metabolize APP. It also cleaves the Notch receptor, releasing the intracellular domain which is a transcription factor that regulates diverse genes; γ secretase also has many other functions (Tolia and De Strooper, 2009; Wolfe, 2009; Carroll and Li, 2016; Wolfe, 2025). PS1-associated secretases are primarily at the cell surface whereas PS2 complexes are mainly in endosomes (Meckler and Checler, 2016). PS1 and PS2 mutations are associated with 20–50% of mutations associated with early-onset AD (Tandon and Fraser, 2002), but the knockout (KO) mice are a deletion, not reflecting the mutations in AD. The mice are useful nevertheless because the PS2 mutations in AD are similar to loss-of-function mutations (Rossi et al., 2021). Remarkably, we found IIS in PS2KO mice, and they were primarily in REM sleep (Lisgaras and Scharfman, 2023a). Thus, many mouse models showed IIS primarily in REM sleep despite very different backgrounds, mutations/deletions, and pathology. The absence of plaque pathology in the PS2KO was especially interesting because it showed again that plaques are not required for IIS. The detection of IIS in PS2KO mice suggest that APP and Aβ may not be the only mechanistic basis for hyperexcitability. Other factors that occur early in AD may also initiate hyperexcitability.

## Cholinergic mechanisms in hyperexcitability

The original “cholinergic hypothesis” suggesting that cholinergic neurons deteriorated in late stages of AD. In support, it has been established that acetylcholine (ACh) is critical to memory, and blocking cholinergic receptors leads to memory impairment (Davies and Maloney, 1976; Jagielska et al., 2025).

The data for the hypothesis were based on assays of choline acetyl transferase (ChAT), the major synthetic enzyme for ACh, and acetylcholinesterase (AChE), the major enzyme that metabolizes ACh. Both enzymes were reduced in patients with end-stage AD. Furthermore, ChAT activity and ACh release were decreased. It should be noted however, that several years later, the hypothesis was reconsidered because new data showed that at early stages of AD, cholinergic deficits were not evident (Davies, 1999).

Based on the idea that early stages of AD may be different from late stages, we considered that cholinergic neurons were overly active early in AD. We hypothesized that the overactivity might ultimately lead to deterioration of cholinergic neurons, in keeping with the idea that at late stages of AD there is a decline in measurements of cholinergic activity. The deterioration of cholinergic neurons might occur due to a direct effect of chronic overactivity, causing metabolic stress. Deterioration might also occur because overactivity might tax the mitochondria or endosomal-lysosomal pathway, which are compromised in AD (Nixon and Rubinsztein, 2024; D'Alessandro et al., 2025). An alternative explanation that does not require cholinergic neuron loss is based on effects of APP and Aβ on cholinergic neurons in rodents (Richter et al., 2018; Hefter et al., 2020). APP caused stimulation of cholinergic neurons whereas increasing Aβ levels decreased cholinergic neurotransmission (Richter et al., 2018; Hefter et al., 2020). Therefore, initially as APP is high but Aβ low, cholinergic neurons may be stimulated, but as Aβ rises later in life, cholinergic neurotransmission may decline.

**Figure 2:**
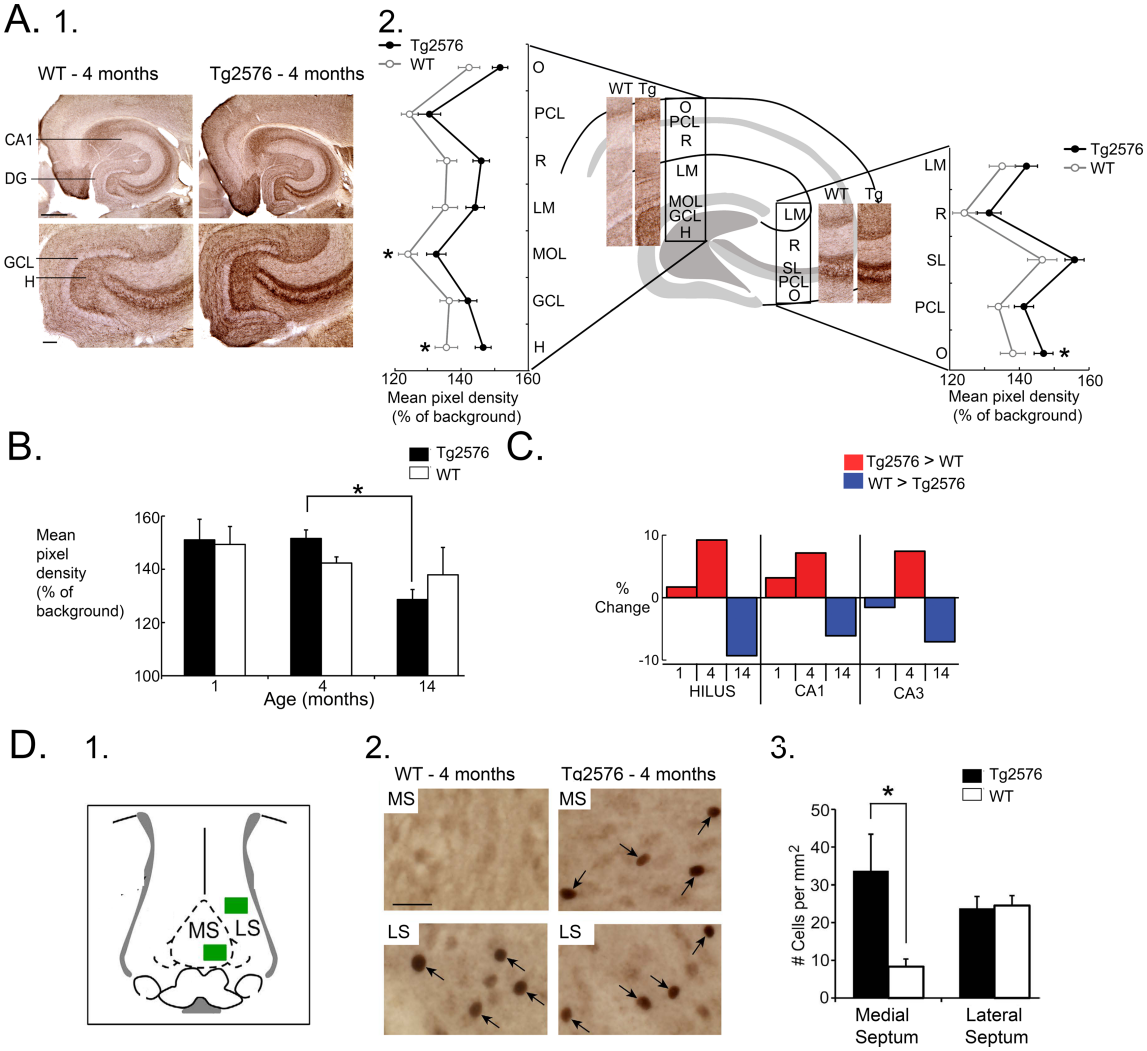
**(A)** 1. Comparison of ChAT-immunoreactivity (ir) in a 4 months-old WT and Tg2576 mouse. Calibration, 100 μm. 2. Mean pixel density of ChAT-ir is plotted for each layer and normalized to background (alveus). O, stratum oriens, PCL, pyramidal cell layer; R, radiatum, LM, lacunosum-moleculare; MOL, molecular layer; GCL, granule cel layer; H, hilus; SL, stratum lucidum. Tg2576 mice had higher ChAT-ir, with post-hoc tests showing signficant differences in the molecular layer, hilus, and stratum oriens of CA3. **(B)** Two-way ANOVA followed by post-hoc tests showed that hilar ChAT was significantly greater in Tg2576 mice at 4 months compared to 14 months. **(C)** Comparison of the % change in ChAT-ir for the hilus, CA1 and CA3 showed Tg2576 mice had higher ChAT levels compared to WT mice at young ages but the opposite at older ages. **(D)** 1. A diagram shows the areas of the medial septum (MS) and lateral septum (LS) where c-Fos-ir cells (arrows) were measured after perfusing animals after 2 h of sleep. 2. At 4 months, there were significantly more c-Fos-ir cells in the MS in Tg2576 mice compared to WT. There were no differences in the LS. * = *p* < 0.05. Adapted from Kam et al. (2016).

This idea was supported by our data showing that ChAT immunocytochemistry was elevated in the 4 months-old Tg2576 hippocampus, rather than depressed, and by 14 months, ChAT had declined (Figures 2A–C; Kam et al., 2016). In addition, a study of ChAT levels in early AD showed it was high in hippocampus, not depressed (DeKosky et al., 2002). Indeed, oligomeric Aβ1-40 and Aβ1-42 increases the catalytic rate of ChAT *in vitro* (Kumar et al., 2018). The idea that high activity of cholinergic neurons could lead to hyperexcitability also was consistent with studies showing that seizures are produced after drugs are used that activate muscarinic receptors (Turski et al., 1983a; Turski et al., 1983b) or increase ACh like organophosphates (Lallement et al., 1998; Todorovic et al., 2012).

As a test of the hypothesis we injected the muscarinic receptor antagonist atropine into Tg2576 mice and, consistent with the hypothesis, found IIS decreased (Kam et al., 2016). A major problem with this approach was that atropine also reduces REM. However, we found that IIS were also decreased in NREM sleep, so we could circumvent the issue simply by turning our attention to NREM sleep. To obtain more evidence for the hypothesis we asked if cholinergic neurons were overactive in young Tg2576 mice. For this purpose, we took advantage of c-Fos, a marker of neurons that have increased neuronal activity. We presumed the increased activity of cholinergic neurons would occur primarily in sleep, especially REM, so we euthanized animals after a 2 h period of sleep. We allowed animals to be in their home cages and spontaneously explore, groom, eat, or sleep. When they slept they were intermittently observed to confirm they were asleep. We allowed mice to be asleep for 2 h because studies of other mice with video and EEG showed that when mice began to sleep, they passed through REM before 2 h had passed.

Since c-Fos protein expression reflects the preceding hrs of neuronal activity (Lara et al., 2022), we perfused them after the 2 h of sleep. We examined c-Fos protein expression in the medial septal neurons for many reasons. First, the medial septum forms the major cholinergic input to the hippocampus. Second, the degeneration of cholinergic neurons in AD has mainly been noted in the medial septum and its neighbor, the nucleus of the diagonal band (Martinez et al., 2021). Third, medial septal neurons are involved in the complex circuitry that is involved in REM sleep, although other cholinergic nuclei are also important (Watson et al., 2010). We quantified c-Fos-expressing neurons in the medial septum because it is much easier to define the borders of the medial septum than the nucleus of the diagonal band, allowing us to quantify all cells within the medial septum with more confidence than the diagonal band. The results showed that the young Tg2576 mice had more c-Fos-expressing neurons in the medial septum than WT mice, and by comparison, the lateral septum showed no differences between Tg2576 and WT (Figure 2D; Kam et al., 2016). It is important to note, however, that there was no double-labeling of c-Fos-expressing medial septal neurons with cholinergic markers, so the medial septal neurons could have been cholinergic or one of the other two medial septal cell types, glutamatergic or GABAergic.

Additional recordings provided more support for the hypothesis that activity in the medial septal cholinergic neurons of young Tg2576 mice contributed to IIS. For these recordings silicon probes were inserted into the hippocampus. Recordings were made in the hippocampus because of the evidence that ChAT was elevated there in young Tg2576 mice, as well as other evidence from our initial recordings of IIS with electrodes, when IIS often appeared to start in the hippocampus (Kam et al., 2016).

**Figure 3:**
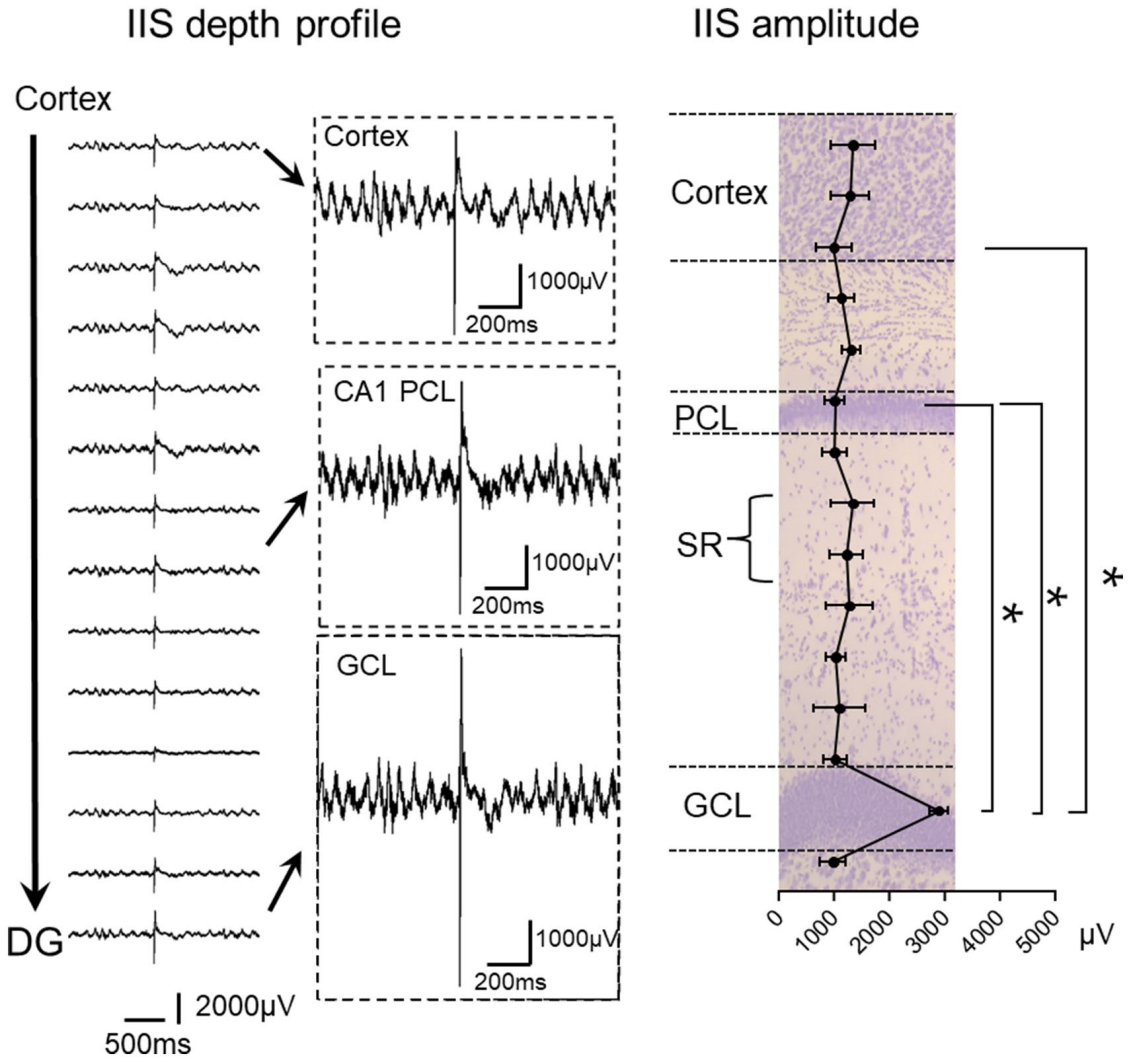
Left: IIS depth profile in a Tg2576 mouse. Recordings are shown from the deep layers of the cortex to the granule cell layer (GCL) of the dentate gyrus (DG). Insets show expanded records, illustrating the greater IIS amplitude in the GCL compared to the CA1 pyramidal cell layer (PCL) and deep layers of cortex. Right: IIS amplitude is plotted for Tg2576 mice showing that there were significantly larger IIS amplitudes in the GCL than other areas (RMANOVA followed by post-hoc tests, * = *p* > 0.05). SR, stratum radiatum. The plot is superimposed on a Nissl stain showing the cell layers in purple. Adapted from Lisgaras and Scharfman (2023b).

The silicon probe recordings were extremely useful because they showed the activity corresponding to IIS in each layer of the cortex over the hippocampus, throughout area CA1, and throughout the dentate gyrus (Figure 3; Lisgaras and Scharfman, 2023a). We were surprised to find that the IIS was largest in the cell layer of the DG (Figure 3; Lisgaras and Scharfman, 2023a). We initially used Tg2576 mice and then confirmed the findings with Ts65Dn and PS2 KO mice (Lisgaras and Scharfman, 2023a).

**Figure 4:**
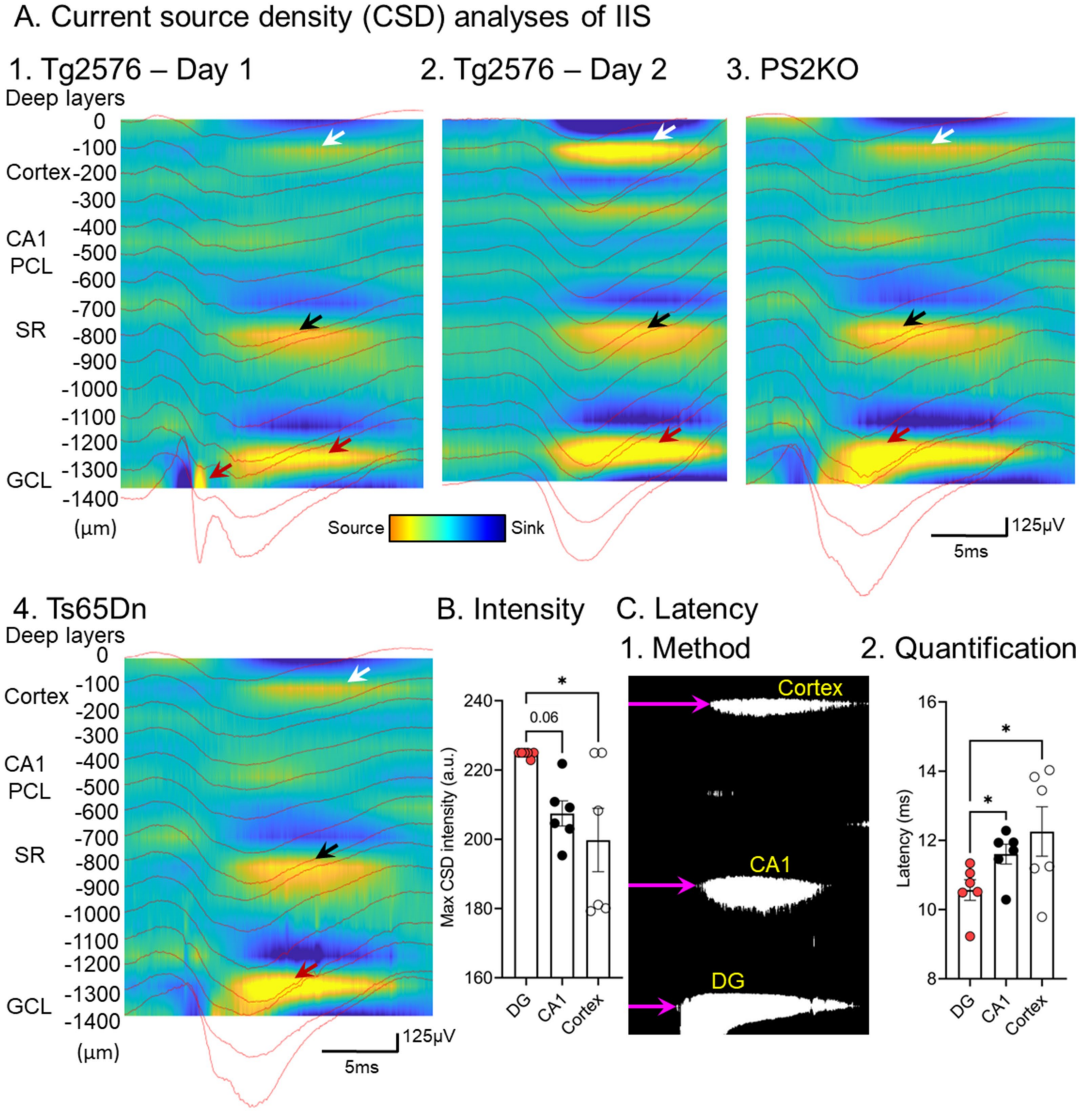
**(A)** Current source density analysis of IIS from 1. A Tg2576 mouse; 2. the same mouse a day later; 3. a PS2KO mouse; 4. a Ts65Dn mouse. The current sources are marked by arrows. In the DG the current sources are greatest (red arrows) compared to the sources in stratum radiatum (black arrows) and the deep layers of cortex (white arrows). **(B)** Quantification of intensity in different Tg2576 mice shows a significantly elevated source intensity in the DG relative to cortex and a trend for greater DG intensity than CA1 (one-way ANOVA followed by Tukey post-hoc tests, * = *p* > 0.05). **(C)** 1. Quantification of latency used a binary image of the CSD and measured the onset of the record to the start of the source (purple arrow). 2. The results showed that the latency was shortest for the DG source (one-way ANOVA followed by Tukey post-hoc tests, * = *p* > 0.05). From Lisgaras and Scharfman (2023b).

Next, we determined the sinks and sources corresponding to the IIS, which would localize the afferent inputs that were responsible for initiating IIS. The current source density (CSD) analysis showed several potential areas of synaptic input might be responsible for triggering IIS (Lisgaras and Scharfman, 2023a). One was in the inner molecular layer (IML) of the dentate gyrus, the layer containing the proximal dendrites of the principal cells, the granule cells (GCs; Figure 4; Lisgaras and Scharfman, 2023a). Another was stratum radiatum, where the apical dendrites of area CA1 pyramidal cells are located (Figure 4). A third was the deep layers of the cortex above the hippocampus (Figure 4; Lisgaras and Scharfman, 2023a).

Remarkably, CSDs were similar from day to day and across mouse models (Figure 4; Lisgaras and Scharfman, 2023a), suggesting similar mechanisms. To understand which of the potential afferents were most important, we quantified the onsets of each source. The sources started in the IML, then were followed milliseconds later by stratum radiatum, and finally the cortex (Figure 4; Lisgaras and Scharfman, 2023a). These data suggested that afferents to the IML trigger the IIS in granule cells, which then trigger activity in area CA3 by the granule cell axons to area CA3. Then CA3 pyramidal cells excite CA1 by the projection of CA3 pyramidal cells to CA1, which ends in stratum radiatum. CA1 then sends its output to cortex. Thus, the trisynaptic circuitry of the hippocampus could explain the sequence.

The finding that the IML might be the site of synaptic input that initiates IIS was interesting because it is a location where cholinergic neurons innervate the dentate gyrus. Thus, acetylcholinesterase staining is greatest there in humans and monkeys (Bakst and Amaral, 1984; Green and Mesulam, 1988). In the rat, cholinergic innervation is also prominent in the IML (Frotscher and Leranth, 1985). Furthermore, muscarinic receptors are dense in the IML (Rouse and Levey, 1997).

With this in mind, we hypothesized that the medial septal cholinergic neurons triggered IIS by releasing ACh in the IML. To test that hypothesis, we used Tg2576 mice with Cre recombinase in neurons that express ChAT (ChAT: Tg2576 mice) and an adeno-associated virus (AAV) virus that encodes the inhibitory protein hM4Di, a designer drug acting on designer receptors (Dreadd). After injecting the virus in the medial septum, hM4Di is expressed in the cholinergic neurons of the medial septum, which we confirmed using a construct with a fluorescent tag on hM4Di (mCherry). EEG electrodes were implanted during the same surgery. Three weeks later, after viral expression, systemic injection of the Dreadd activator clozapine-N-oxide (CNO) activates hM4Di in in medial septal neurons, inhibiting their firing by opening G-protein coupled inwardly rectifying potassium channels (Pei et al., 2008). Comparisons were made to saline instead of CNO, or Tg2576 mice with CNO but not hM4Di.

**Figure 5:**
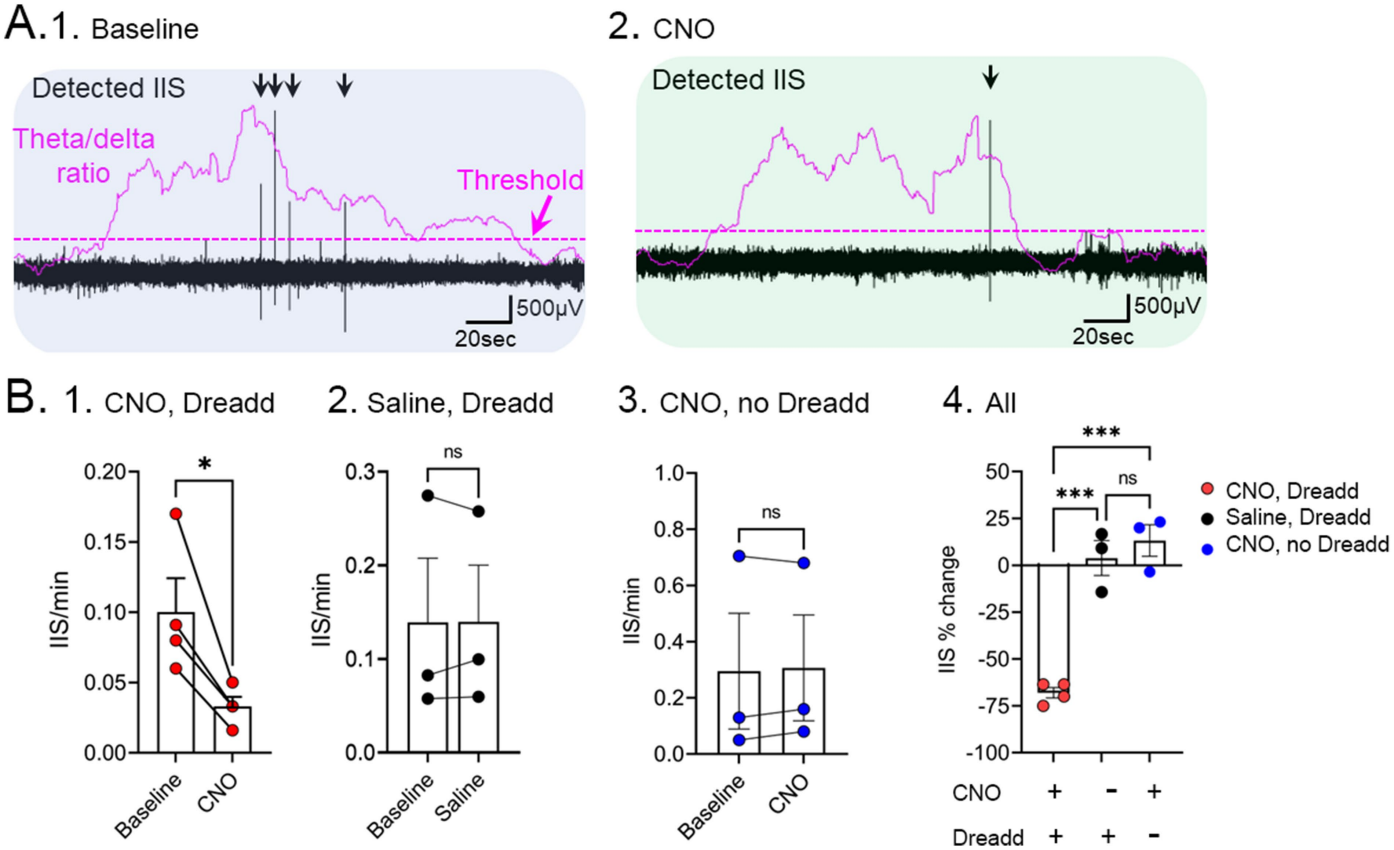
Inhibition of IIS by chemogenetic silencing of medial septal cholinergic neurons in Tg2576 mice. **(A)** Chat-Cre + mice were crossed to Tg2576 mice and Cre-dependent AAV encoding the inhibitory Dreadd hM4Di was injected into the medial septum. 1. A baseline was recorded showing IIS (black arrows) during sleep. The theta/delta ratio shows an elevation during REM. A dotted line illustrates the threshold used to differentiate increased theta/delta ratio, consistent with REM sleep. 2. After injection of CNO, there was a reduction of IIS. **(B)** Comparisons of 1. Cre + Tg2576 mice injected with AAV encoding hM4Di before and after CNO shows a significant reduction in IIS/min (t-test, * = *p* < 0.05). 2. Cre + mice that received the Dreadd but saline instead of CNO showed no significant differences (ns). 3. Tg2576 mice that did not receive the Dreadd but did receive CNO showed no significant differences. 4. Comparison of all experimental groups show a significant % reduction in IIS only for the group that had CNO and Dreadd. Adapted from Lisgaras and Scharfman (2023b).

The results showed the IIS were reduced by CNO but saline had no significant effect (Figure 5; Lisgaras and Scharfman, 2023a). CNO had no effect in Tg2576 mice without hM4Di (Figure 5; Lisgaras and Scharfman, 2023a). These data provided solid support for the idea that the media septal cholinergic neurons were critical to IIS generation in young Tg2576 mice. They also supported the hypothesis that this cholinergic input is overly active early in life, so inhibiting it reduces IIS. In the future we will test if increasing cholinergic input using the excitatory Dreadd hM3Dq can increase IIS.

It should be noted that more complex circuitry could underlie IIS based on the regulation of septal GABAergic neurons by septal cholinergic neurons (Gu and Yakel, 2022). Muscarinic receptors on the septal GABAergic neurons are activated by ACh release from septal cholinergic neurons. The activated septal GABAergic neurons project to hippocampal GABAergic neurons and depress their activity, leading to more firing of principal cells. In light of this, any glutamatergic input to the IML, such as the input from mossy cells, could lead to more firing than usual in GCs. Furthermore, there is a direct septohippocampal cholinergic input to mossy cells that could increase their excitability as the GABAergic neurons from the septum inhibit adjacent GABAergic neurons (Leranth and Hajszan, 2007).

One of the questions that has not been addressed is why there might be increased activity of cholinergic neurons in young Tg2576 mice. One explanation is based on the data showing that low levels of oligomeric Aβ increase excitability of cholinergic neurons. The data used recordings from dissociated cholinergic neurons of the nucleus of the diagonal band or organotypic cultures of basal forebrain. Oligomeric Aβ reduced a calcium-activated K^+^ current (I_C_) and delayed rectifier K + current (I_A_). The reduction in K^+^ currents led to increased neuronal firing. Furthermore, effects were on cholinergic, not GABAergic neurons (Jhamandas et al., 2001; Kar et al., 2004; George et al., 2021) but some effects were found on medial septal rather than all cholinergic neurons (George et al., 2021). A caveat with these data is that they were from reduced preparations which may not be generalized to *in vivo* conditions. Moreover, synthetic oligomeric Aβ may not be the same as oligomeric Aβ in vivo, and effects may depend on the peptide, e.g., Aβ1-40 vs. Aβ1-42.

It is also important to consider a role of p75 neurotrophin receptors (p75^NTR^) since they are enriched in cholinergic nerve terminals (Hartig et al., 1998; Mufson et al., 2003) and bind Aβ (Xia et al., 2016). Most research has focused on a role of p75^NTR^ in the degeneration of cholinergic neurons in AD because p75^NTR^ triggers cell death (Coulson et al., 2009). However, p75^NTR^ activation leads to numerous other effects, and one of them is to activate G-protein inward rectifying K^+^ channels (GIRK channels) which lead to elevation in extracellular K^+^ (Coulson et al., 2008). Indeed, this elevation in K^+^ appears to be critical to the process leading to cell death (Coulson et al., 2008). The extracellular rise in K^+^ could contribute to increased excitability either of cholinergic terminals, increasing ACh release, or postsynaptic neurons, leading to their depolarization.

High levels of APP could have many effects that increase cholinergic neuronal activity independent of Aβ. At low levels APP increases cholinergic neurotransmission due to interactions with α7 nicotinic receptors, which are enriched in cholinergic neurons. As APP levels rise and Aβ concentrations reach low levels, Aβ antagonizes these effects of APP (Richter et al., 2018; Hefter et al., 2020). This is an intriguing set of observations because it suggests how early in life APP may increase cholinergic activity and later in life as Aβ levels rise, cholinergic activity would decline.

Although unlikely to play a role in the mouse models we have used, tau and APOE4 genotype may contribute to early hyperexcitability of the cholinergic neurons in AD. For tau, some studies suggest that hyperphosphorylated and aggregated tau disrupts cholinergic neurons in the basal forebrain, leading to their degeneration (Schliebs and Arendt, 2011; Rajmohan and Reddy, 2017). However, early AD still needs to be studied. Effects of APOE genotype on excitability are complex (Konings et al., 2021) and few studies have asked how APOE genotype influences cholinergic activity. One *in vitro* study showed that neurons with APOE-ε4 expression had reduced acetyl-CoA, leading to increased ACh synthesis and increased levels of extracellular ACh (Piccarducci et al., 2023). If one can generalize from those data, the results would be consistent with the idea that APOE-ε4 genotype places individuals at risk for hyperexcitability by driving ACh synthesis and release. However, in the same study, APOE-ε4 expression had many additional effects that might increase or decrease excitability. Furthermore, the majority of APOE is in astrocytes, not neurons (Konings et al., 2021).

Another question is why IIS would begin in the dentate gyrus given the cholinergic neurons of the medial septum innervate many areas of hippocampus and cortex as well as the dentate gyrus. One reason is the mossy cells, a glutamatergic hilar cell type that has no clear analog in other hippocampal subfields or cortical circuits. The mossy cell has the ability to synchronize granule cells through its en passant synapses and widespread projection to granule cells across the septotemporal axis and bilaterally (Buckmaster et al., 1992; Buckmaster et al., 1996) and the GABAergic neurons from the septum inhibit GABAergic neurons of the dentate gyrus (Freund and Antal, 1988). Mossy cells receive direct input from medial septal cholinergic neurons (Deller et al., 1999) and are excited by them (Hofmann and Frazier, 2010). One could argue that mossy cells might not excite granule cells very much because mossy cells also excite local GABAergic neurons (Scharfman, 2016). However, this may not be true in AD because GABAergic neurons in the dentate gyrus are vulnerable in AD, especially the so-called HIPP cells that co-localize neuropeptide Y (Chan-Palay et al., 1986; Loreth et al., 2012; Albuquerque et al., 2015) and somatostatin (Rossor et al., 1980; Chan-Palay, 1987; Ramos et al., 2006). It should be noted, however, that some mouse models appear to show GABAergic neurons in the dentate gyrus exhibit increased peptide expression (Palop et al., 2011) which could occur because hyperexcitability increases NPY protein in dentate gyrus GABAergic neurons (Marksteiner et al., 1989). Additional contributions to activity of granule cells in sleep may be due to the depression of GABAergic neurons by subcortical afferents that become active in sleep (Watson et al., 2010). Therefore, the particular circuitry and susceptibility of dentate gyrus may predispose it to generate IIS in sleep.

**Figure 6:**
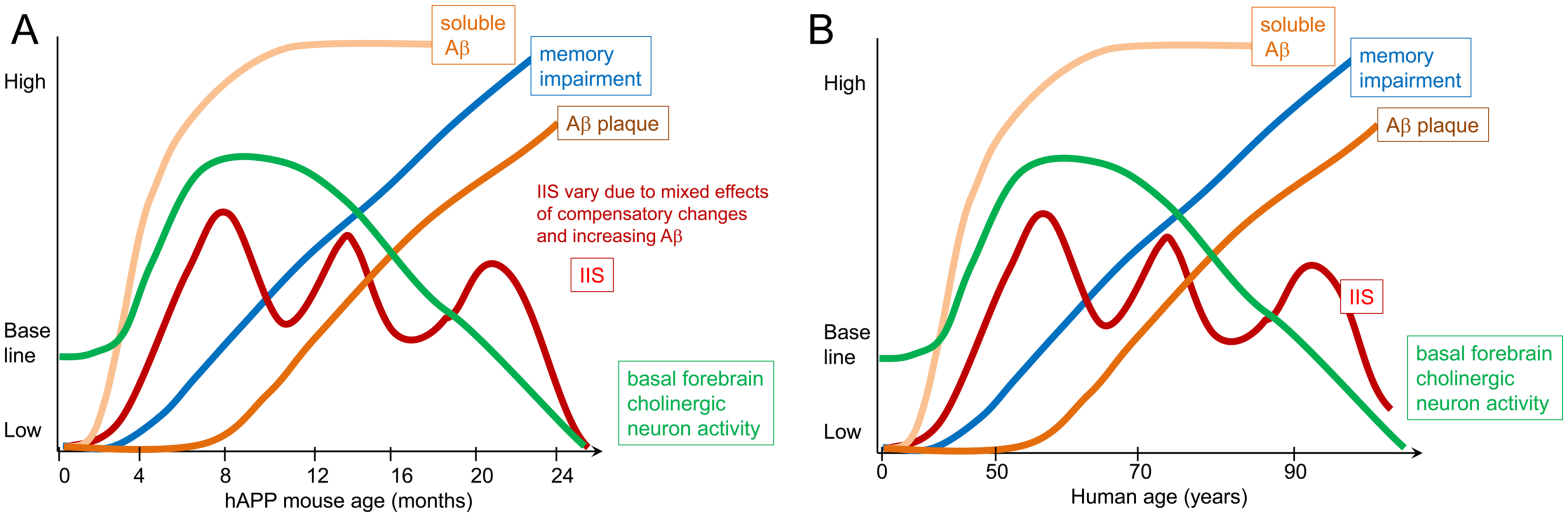
Schematic of the hypothesis. **(A)** Tg2576 mouse model. By 1 month of age there are IIS that occur spontaneously, suggesting hyperexcitability. Soluble Aβ is present but plaque does not occur until 6 months of age. Memory impairment develops by 3–4 months of age, similar to the age when IIS rates peak. Subsequently IIS rates become variable, possibly because other deficits occur and are followed by transient compensatory changes. Regarding the role of acetylcholine, there is evidence that the medial septal cholinergic neurons are overly active in the first months of life and lead to IIS because inhibiting the cholinergic neurons reduces IIS. In contrast, in old age there is a decline in cholinergic function. Importantly, the literature differs regarding the degree cholinergic neurons degenerate, simply lose function but remain viable, and whether they become hypoactive (Mufson et al., 2003; Mufson et al., 2016). **(B)** In humans, epileptiform activity and seizures can occur at early stages but some individuals may not show signs of epileptiform activity or seizures, which may be due to a lack of recording during sleep, low frequency of events, or lack of recording from deep brain structures (Lam et al., 2017). Also at early stages there is evidence that cholinergic neuron markers such as ChAT increase although how much cholinergic activity increases, where it does, and at what age is not clear. Late AD stages are characterized by basal forebrain cholinergic neurodegeneration. The timing of memory impairment varies in the literature, and the most sensitive tests of memory may not be tested, making it unclear exactly when memory impairment begins relative to IIS and cholinergic changes. Similarly, it remains to be clarified exactly what when soluble Aβ rises, where it does, and what are its initial effects. Thus disease progression can mainly be divided into an early and late phase that varies with each patient, with an early increase in neuronal activity likely, and changes in cholinergic neurons. Late in the disease manifestations of hyperexcitability such as IIS need more examination. However, most individuals exhibit cholinergic dysfunction and/or degeneration.

Figure 6A shows a schematic of what the data suggest in mice. Early in life, long before signs of cognitive impairment, we suggest that IIS occur in sleep and start to disrupt memory consolidation, as well as disrupt clearance of waste products like Aβ. With time hyperexcitability becomes more common, and this leads to increasing impairments. At older ages there is a deterioration of cholinergic function, presumably because chronic hyperexcitability leads to a buildup of toxic waste products in the cell, or other effects of chronic metabolic stress. However, hyperexcitability persists. The persistence may be due to additional pathology that occurs as Aβ plaques develop such as increasing impairments in GABAergic neurons (Verret et al., 2012; Martinez-Losa et al., 2018). Hyperexcitability is variable, which could be due to GABAergic deficits increasing and loss of spines and glutamatergic synapses decreasing excitability.

In Figure 6B we diagram what might occur in humans with AD. Although the diagram is analogous to Figure 6A, it is likely that all patients with AD will not follow the same trajectory. Another important caveat with the diagram is that neurofibrillary tangles are not in the diagram. Therefore, the schematic is only a hypothesis for what may occur based on the studies of mouse models.

It should be noted that in the mouse models, memory impairment has been shown before or at the same time as amyloid deposition. However, in humans, amyloid deposition can be earlier or later than cognitive defects. Importantly, it may depend on the task used to test memory and the type of Aβ measurement (plasma, neuroimaging, plaque defined by Thioflavin-S, etc.) and the type of Aβ (Aβ1-40, Aβ1-42, oligomeric, etc.). Regarding tasks, we hypothesize that the tasks which are most sensitive depend on the entorhinal cortex and dentate gyrus based on our finding that these tasks identified early impairments in mice that had not previously been recognized (Duffy et al., 2015). Tasks which depend on the dentate gyrus also appear to be useful in human studies (Stark et al., 2013).

## Choline supplementation

The data support the hypothesis that a problem in AD is early overactivity of cholinergic neurons. In addition, infusion of oligomeric Aβ into the cortex of rodents appears to interfere with choline uptake *in vitro* (Parikh et al., 2014). Mutant forms of APP such as APPSwe appear to prevent choline reuptake relative to WT APP, at least in vitro (Cuddy et al., 2015). If this is true, presynaptic stores of choline may be depleted early in the disease. Therefore, supporting choline levels might be therapeutic if possible. Indeed, in many pathological conditions, choline supplementation has been restorative (Bottom et al., 2020; Ren et al., 2024) although whether it is due to cholinergic neuron support or other beneficial effects of choline is not clear.

One method for increasing dietary choline that has shown benefits is maternal supplementation with choline. In the diet, choline is a common nutrient found in foods such as eggs, meat, fish and some vegetables (Blusztajn and Mellott, 2012). Although it is critical for ACh synthesis, choline is also essential to neural development (Blusztajn and Mellott, 2012; Derbyshire and Obeid, 2020), and has several other functions (Blusztajn et al., 2017). As a methyl donor, it has epigenetic actions (Blusztajn and Mellott, 2012; Blusztajn et al., 2017; Socha et al., 2024). In the brain, choline is important to membrane phospholipid synthesis as a precursor to phosphatidyl choline (Zeisel and da Costa, 2009; Blusztajn et al., 2017).

We are not the first to consider dietary choline for AD. There is a growing literature about its potential for AD. Low levels of circulating choline are associated with adverse outcomes in patients with AD and mouse models (Judd et al., 2023). Increasing choline perinatally improved outcomes in APP*^NL-G-F^* mice (Bellio et al., 2024). Amyloid levels were reduced in APP/PS1 mice by MCS (Mellott et al., 2017). Although the results of choline supplementation late in AD are small (Amenta et al., 2001), prenatal or perinatal supplementation appears to have benefits (Velazquez et al., 2020). Moreover, in the Ts65Dn model, maternal choline supplementation improved early pathology of cholinergic neurons (Kelley et al., 2016; Gautier et al., 2023; Gautier et al., 2024) and cognition (Moon et al., 2010; Strupp et al., 2016) and has been suggested for mothers (Powers et al., 2021).

**Figure 7:**
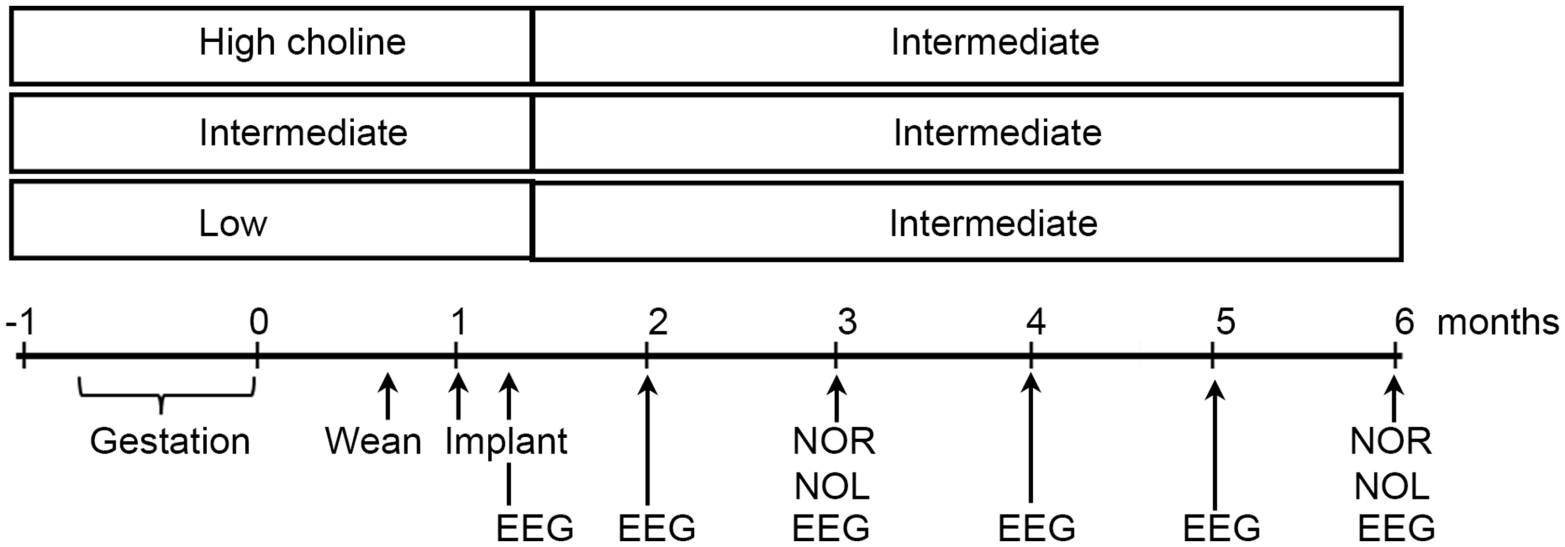
Experimental timeline. Three diets were compared, one relatively high in choline chloride, another standard rodent chow, and a diet relatively low in choline. Diets were administered from the start of mating until weaning. Then all mice were fed the standard diet. Animals were implanted with 4 electrodes at 1 month of age and recordings made at 5 weeks, 2, 3, 4, 5, and 6 months. At 3 and 6 months of age, novel object location (NOL) and novel object recognition (NOR) were tested. At 6 months, mice were perfused and immunocytochemistry was performed with antibodies to ΔFosB and NeuN.

In light of these studies, we hypothesized that choline supplementation would prevent hyperactivity in Tg2576 mice. We chose to give mice three diets: the one that we had been using, a standard rodent chow, and two others that were exactly matched except for choline chloride (Figure 7). The supplemented diet provided 5.0 g choline chloride/kg chow. The low choline diet that was used for direct comparison had 1.1 g/kg. The standard diet had 2.0 g/kg. It is important to point out that almost all investigators who use the supplemented diets in rodents use 5.0 g/kg and almost all who use a matched diet with lower choline use 1.0 g/kg choline. This makes results of different rodent studies easier to compare, except that some studies start the diet in the middle of gestation, and others start the diet at other times. Also, some laboratories end the diet at birth and others end it much later. We chose to start the diets during mating, approximately 1 week before the onset of pregnancy, and diets were switched to the standard chow after weaning. Our goal was to maximize the opportunity for an effect by increasing the temporal window when mothers had a supplemented diet. It should be noted that the 1.1 g/kg diet is considered adequate by the Institutes of Medicine (IOM, 1998), so we use the phrases “low” choline diet and “high” choline diet only as relative terms.

In our studies, mice were implanted with electrodes at 1 month and then recordings began a week later. They were recorded at 2,3,4,5, and 6 months of age, and then were perfused. Immunocytochemistry was used to assay ΔFosB as a marker of chronically elevated activity. We were curious if the choline supplemented diet reduced activity both by EEG and ΔFosB.

**Figure 8:**
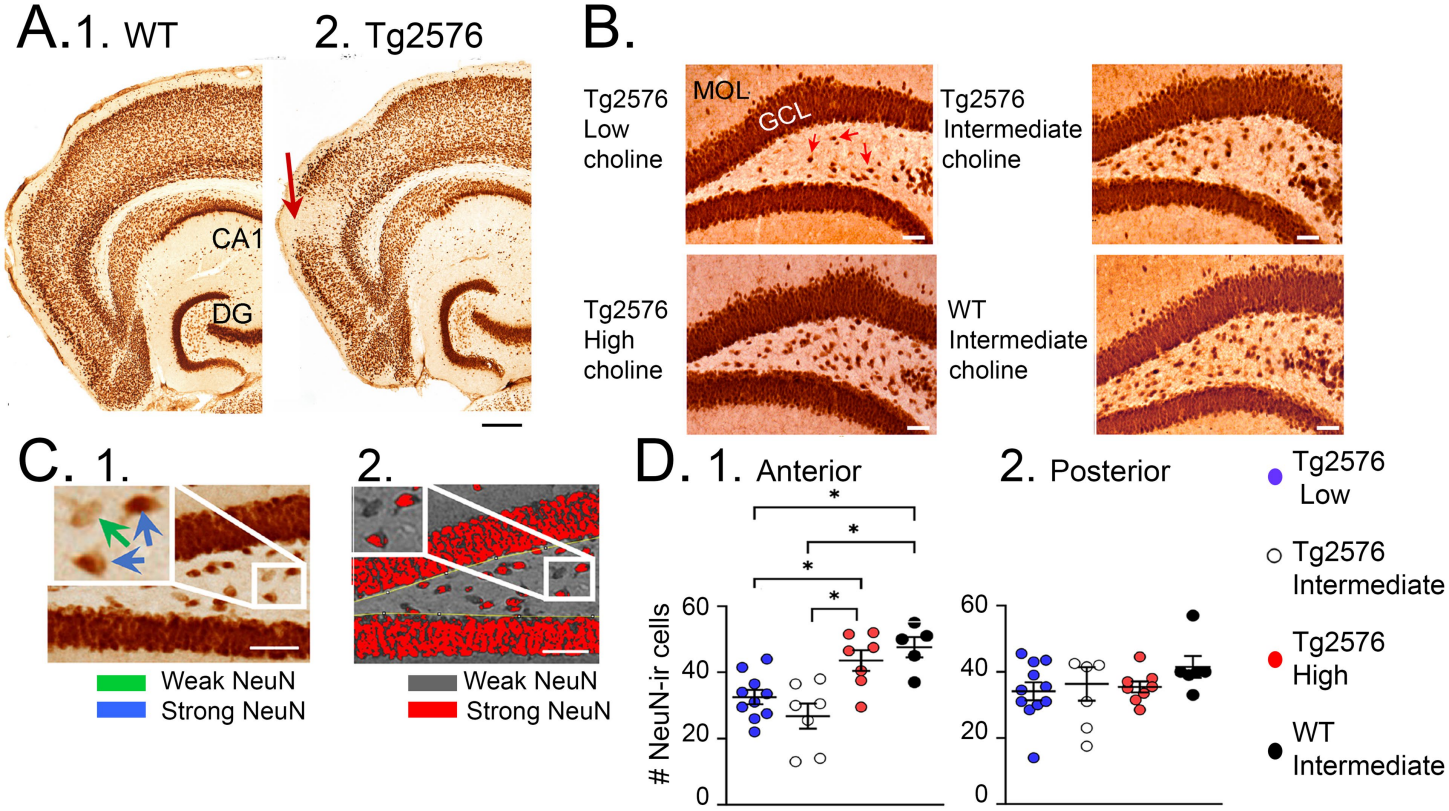
NeuN rescue by a perinatal high choline diet. **(A)** NeuN is an antigen that is dephosphorylated by pathological conditions, so that the antibody to NeuN fails to recognize the NeuN antigen and immunoreactivity is lost. Examples of NeuN loss (arrows) are shown for a WT (1) and Tg2576 mouse (2). Calibration, 200 μm. **(B)** Comparisons of dentate gyrus NeuN in a Tg2576 mouse treated with the low choline diet; the intermediate diet; the high choline diet; and a WT mouse treated with the intermediate diet. Calibration, 50 μm. Red arrows point to hilar cells with weak NeuN immunoreactivity. **(C)** 1. Quantification of hilar NeuN protein used a thresholding method to make those cells with strong expression (blue arrows in the inset) red and those with weak expression (green arrow) grey. 2. Results of thresholding. **(D)** 1. Results showed that the mice that had the high choline diet had more NeuN-ir cells than the mice fed the low (*p* = 0.046) or intermediate (*p* = 0.003) diets. NeuN-ir cells in mice fed the high choline diet were restored to numbers in WT mice (these 2 groups were not different, post-hoc tests, *p* = 0.827). 2. The results were not significant for posterior dentate gyrus. **(A)** is adapted from Duffy et al. (2015); **(B,C)** are adapted from Chartampila et al. (2024).

We also studied NeuN, a marker of neurons. Instead of its typical use as a marker of neurons, we used it as a marker of oxidative stress. Thus, the NeuN protein becomes dephosphorylated in pathological conditions and the antibody no longer recognizes the antigen (Lind et al., 2005). The result is a loss of NeuN staining, but it is because of oxidative stress, not neuronal loss. Dephosphorylation of NeuN occurs in many pathological conditions such as ischemia (Unal-Cevik et al., 2004) and loss of neurotrophic support (Duffy et al., 2011) and we have documented it in the Tg2576 mouse entorhinal cortex and frontal cortex (Duffy et al., 2015). In the present study of choline supplementation we studied neurons that lose NeuN in the hilus (Figure 8). They develop soluble Aβ as early as 1 month (Alcantara-Gonzalez et al., 2021), probably explaining why they lose NeuN immunoreactivity.

**Figure 9:**
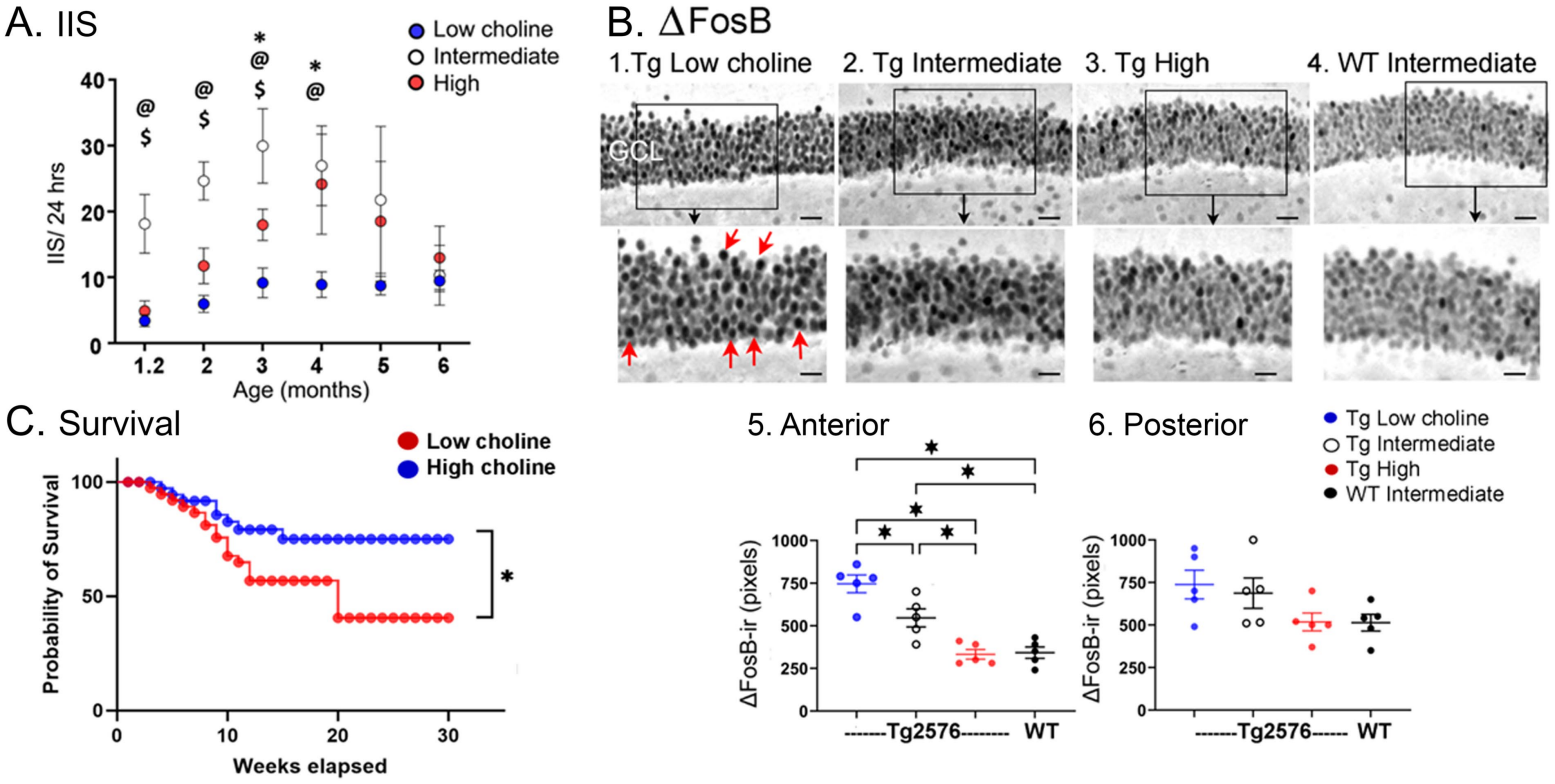
**(A)** IIS rates are shown as Tg2576 mice aged. The highest rates were Tg2576 mice that had been fed the intermediate diet perinatally. Significant differences were found among the mice fed the different diets until 5 months of age, when IIS became extremely variable. Two-way ANOVA followed by Tukey post-hoc tests, @ = *p* < 0.05, intermediate vs. low; $ = *p* < 0.05, intermediate vs. high; * = *p* < 0.05, low vs. high. **(B)** Tg2576 mice fed the lower choline perinatal diets (1,2) had elevated ΔFosB ir in the GCL of the dentate gyrus, suggesting increased excitability. The Tg2576 mice fed the high choline diet 3. showed ΔFosB-ir similar to the WT mice fed the intermediate diet 4. The areas surrounded by the boxes are shown at higher power in the lower panel. Red arrows point to strongly expressing GCs. Calibrations, 20 μm; 10 μm. Differences were significant when quantified in anterior (1) but not posterior dentate gyrus (2), probably because of more variability in posterior sections. However, a similar pattern was found, with the high choline diet and WT mice showing the least ΔFosB-ir. **(C)** Mice fed the low choline diet had a signficantly higher mortality compared to mice fed the high choline diet (Log-rank (Mantel-Cox) test, *p* = 0.021) suggesting their high excitability led to seizures and sudden death in seizures. Indeed, most mice were found in positions consistent with a severe tonic–clonic seizure. Adapted from Chartampila et al. (2024).

Compared to the standard diet, the mice fed the high choline diet showed fewer IIS (Figure 9; Chartampila et al., 2024). After 6 months, high choline treated mice had reduced ΔFosB in the GC layer (Figure 9; Chartampila et al., 2024). These data suggested that maternal choline supplementation reduced hyperexcitability and improved behavior. It also reduced the loss of NeuN in hilar neurons (Figure 7; Chartampila et al., 2024).

On the other hand, the animals that were fed a relatively low choline diet had several adverse effects. They showed the highest excitability based on ΔFosB in the GCL (Figure 9A; Chartampila et al., 2024), suggesting that low choline impairs, and high choline protects against hyperexcitability. They also showed the most mortality (Figure 9C; Chartampila et al., 2024), again suggesting that low choline has adverse effects and high choline improves overall health. The ability of mice fed the low choline diet to perform novel object location was impaired, like mice fed the standard diet; even WT mice were impaired if they were fed the low choline diet (Chartampila et al., 2024). The extent of NeuN loss was greatest in mice fed the low choline diet, whereas in the mice fed the high choline diet it was best (Figure 8; Chartampila et al., 2024). These data suggested that the low choline diet had several adverse effects.

We expected mice fed the low choline diet would have the most IIS because ΔFosB levels were so high. However, IIS were reduced in mice fed the low choline diet (Figure 9B; Chartampila et al., 2024). We speculate that these mice had few IIS because they were in poor health. Thus, they died prematurely. One reason they could have been died prematurely is that choline is important to normal function of the brain and peripheral organs. Another sign of poor health was impaired memory which occurred even in the WT mice.

It should be noted that most investigators do not consider the low levels of choline that we provided with the low choline diet were inadequate, as mentioned above. Therefore it is not clear why mice would have been adversely affected by the diet we call low in choline. On the other hand, the background strain of the Tg2576 mice is unusual (SBJL) and there is a vulnerability in this strain due to high blood pressure, high heart rate, myopathy, death by 14–20 months (relatively early) and risk of sarcoma (Festing, 1998). Our research suggests that MCS is able to ameliorate many impairments of the Tg2576 mice, and also raises the novel idea that previously approved minimum levels of dietary choline may be low in some susceptible populations.

## Summary

We have briefly reviewed the reason why it is important to study early stages of AD and hyperexcitability in humans and mouse models. Then we presented the idea that IIS are a more common finding rather than seizures. On the other hand, subsets of patients with AD and some mouse models do have robust seizures and more research is warranted to understand the individuals with frequent seizures. Studies are needed to clarify not only if IIS, IEDs, and seizures occur, but when they do relative to memory impairment and neuropathology. These studies would be especially fruitful if they would involve EEG evaluation during sleep at early ages, since it could become a screening tool to identify at-risk individuals. However, to become a screening tool the early evaluations would have to be followed by subsequent evaluations as an individual is first diagnosed and then worsens. These types of long-term studies are challenging.

Next, we showed that in 5 mouse models of AD, including one model of Down syndrome, IIS occur primarily in sleep and therefore could have adverse effects on memory and toxic peptide levels by disrupting normal sleep functions involved in memory consolidation and glymphatic clearance of waste products. Through an analysis of the mice we developed the hypothesis that epileptiform activity is generated in the dentate gyrus by inputs to the proximal dendrites, and the medial septal cholinergic input appears to be very important. We suggest the novel hypothesis that early in life there is cholinergic overactivity that drives the dentate gyrus to produce IIS, and the IIS contribute to progression of the disease. Although unproven as yet, the hypothesis is supported by the presence of IIS in sleep before memory impairment and plaque deposition in mice. Also in support of the hypothesis, systemic muscarinic blockade and selective inhibition of medial septal cholinergic neurons reduced IIS in mice. At late stages, cholinergic neurons are adversely affected, which we presume is in part because of their prior overactivity.

Clearly, a challenge is how to treat the disease. We suggest, as others have before, that prenatal choline supplementation should be considered. Other ways to reduce cholinergic overactivity early in life would be valuable to consider also, although premature at the present time without more information. If possible, one would only want to normalize activity because reducing it too much would be likely to impair memory. We suggested that caution is necessary before use of cholinergic stimulation such as vagal nerve stimulation since it could promote AD instead of improving symptoms. Moreover, in those individuals who do not have a diagnosis of AD but are at risk, such as those with epilepsy, vagal nerve stimulation may be contraindicated. There already has been use of vagal nerve stimulation for epilepsy for a long time, so it might be possible to determine already if vagal nerve stimulation preceded a diagnosis of AD. Cholinesterase inhibitors such as donepezil may have weak effects because at some points in the progression of AD there could already be heightened cholinergic tone. Fortuitously, there are multiple mechanisms contributing to hyperexcitability so even if cholinergic strategies are challenging, there are many other possible therapeutic targets.

## References

[ref1] Albuquerque M. S. Mahar I. Davoli M. A. Chabot J. G. Mechawar N. Quirion R.. (2015). Regional and sub-regional differences in hippocampal GABAergic neuronal vulnerability in the TgCRND8 mouse model of Alzheimer's disease. Front. Aging Neurosci. 7:30. doi: 10.3389/fnagi.2015.00030, PMID: 25852545 PMC4371759

[ref2] Alcantara-Gonzalez D. Chartampila E. Criscuolo C. Scharfman H. E. (2021). Early changes in synaptic and intrinsic properties of dentate gyrus granule cells in a mouse model of Alzheimer's disease neuropathology and atypical effects of the cholinergic antagonist atropine. Neurobiol. Dis. 152:105274. doi: 10.1016/j.nbd.2021.105274, PMID: 33484828 PMC7956160

[ref3] Alldred M. J. Lee S. H. Petkova E. Ginsberg S. D. (2015a). Expression profile analysis of vulnerable CA1 pyramidal neurons in young-middle-aged Ts65Dn mice. J. Comp. Neurol. 523, 61–74. doi: 10.1002/cne.23663, PMID: 25131634 PMC4232465

[ref4] Alldred M. J. Lee S. H. Petkova E. Ginsberg S. D. (2015b). Expression profile analysis of hippocampal CA1 pyramidal neurons in aged Ts65Dn mice, a model of Down syndrome (DS) and Alzheimer's disease (AD). Brain Struct. Funct. 220, 2983–2996. doi: 10.1007/s00429-014-0839-0, PMID: 25031177 PMC4297601

[ref5] Almeida-Filho D. G. Queiroz C. M. Ribeiro S. (2018). Memory corticalization triggered by REM sleep: mechanisms of cellular and systems consolidation. Cell. Mol. Life Sci. 75, 3715–3740. doi: 10.1007/s00018-018-2886-9, PMID: 30054638 PMC11105475

[ref6] Amenta F. Parnetti L. Gallai V. Wallin A. (2001). Treatment of cognitive dysfunction associated with Alzheimer's disease with cholinergic precursors. Ineffective treatments or inappropriate approaches? Mech. Ageing Dev. 122, 2025–2040. doi: 10.1016/S0047-6374(01)00310-4, PMID: 11589920

[ref7] Bakker A. Krauss G. L. Albert M. S. Speck C. L. Jones L. R. Stark C. E.. (2012). Reduction of hippocampal hyperactivity improves cognition in amnestic mild cognitive impairment. Neuron 74, 467–474. doi: 10.1016/j.neuron.2012.03.023, PMID: 22578498 PMC3351697

[ref8] Bakst I. Amaral D. G. (1984). The distribution of acetylcholinesterase in the hippocampal formation of the monkey. J. Comp. Neurol. 225, 344–371. doi: 10.1002/cne.902250304, PMID: 6725649

[ref9] Barbour A. J. Gourmaud S. Lancaster E. Li X. Stewart D. A. Hoag K. F.. (2024). Seizures exacerbate excitatory: inhibitory imbalance in Alzheimer's disease and 5XFAD mice. Brain 147, 2169–2184. doi: 10.1093/brain/awae126, PMID: 38662500 PMC11146435

[ref10] Bellio T. A. Laguna-Torres J. Y. Campion M. S. Chou J. Yee S. Blusztajn J. K.. (2024). Perinatal choline supplementation prevents learning and memory deficits and reduces brain amyloid Aβ42 deposition in APP^NL-G-F^ Alzheimer's disease model mice. PLoS One 19:e0297289. doi: 10.1371/journal.pone.0297289, PMID: 38315685 PMC10843108

[ref11] Bero A. W. Yan P. Roh J. H. Cirrito J. R. Stewart F. R. Raichle M. E.. (2011). Neuronal activity regulates the regional vulnerability to amyloid-β deposition. Nat. Neurosci. 14, 750–756. doi: 10.1038/nn.2801, PMID: 21532579 PMC3102784

[ref12] Bezzina C. Verret L. Juan C. Remaud J. Halley H. Rampon C.. (2015). Early onset of hypersynchronous network activity and expression of a marker of chronic seizures in the Tg2576 mouse model of Alzheimer's disease. PLoS One 10:e0119910. doi: 10.1371/journal.pone.0119910, PMID: 25768013 PMC4358928

[ref13] Bird T. D. Lampe T. H. Nemens E. J. Sumi S. M. Nochlin D. Schellenberg G. D.. (1989). Characteristics of familial Alzheimer's disease in nine kindreds of Volga german ancestry. Prog. Clin. Biol. Res. 317, 229–234, PMID: 2602419

[ref14] Blusztajn J. K. Mellott T. J. (2012). Choline nutrition programs brain development via DNA and histone methylation. Cent. Nerv. Syst. Agents Med. Chem. 12, 82–94. doi: 10.2174/187152412800792706, PMID: 22483275 PMC5612430

[ref15] Blusztajn J. K. Slack B. E. Mellott T. J. (2017). Neuroprotective actions of dietary choline. Nutrients 9:815. doi: 10.3390/nu9080815, PMID: 28788094 PMC5579609

[ref16] Bottom R. T. Abbott C. W. 3rd Huffman K. J. (2020). Rescue of ethanol-induced FASD-like phenotypes via prenatal co-administration of choline. Neuropharmacology 168:107990. doi: 10.1016/j.neuropharm.2020.107990, PMID: 32044264

[ref17] Broer S. Pauletti A. (2024). Microglia and infiltrating macrophages in ictogenesis and epileptogenesis. Front. Mol. Neurosci. 17:1404022. doi: 10.3389/fnmol.2024.1404022, PMID: 38873242 PMC11171130

[ref18] Brooks-Kayal A. R. Raol Y. H. Russek S. J. (2009). Alteration of epileptogenesis genes. Neurotherapeutics 6, 312–318. doi: 10.1016/j.nurt.2009.01.019, PMID: 19332325 PMC2700027

[ref9001] Brown R. Lam A. D. Gonzalez-Sulser A. Ying A. Jones M. Chou R. C.. (2018). Circadian and brain state modulation of network Hyperexcitability in Alzheimer’s disease. eNeuro. 5:1–16. 10.1523/ENEURO.0426-17.2018 PMC5956746 29780880

[ref19] Buckmaster P. S. Strowbridge B. W. Kunkel D. D. Schmiege D. L. Schwartzkroin P. A. (1992). Mossy cell axonal projections to the dentate gyrus molecular layer in the rat hippocampal slice. Hippocampus 2, 349–362. doi: 10.1002/hipo.450020403, PMID: 1284975

[ref20] Buckmaster P. S. Wenzel H. J. Kunkel D. D. Schwartzkroin P. A. (1996). Axon arbors and synaptic connections of hippocampal mossy cells in the rat in vivo. J. Comp. Neurol. 366, 271–292. doi: 10.1002/(sici)1096-9861(19960304)366:2<270::aid-cne7>3.0.co;2-2, PMID: 8698887

[ref21] Bugiani O. (2000). FTDP-17: phenotypical heterogeneity within P301S. Ann. Neurol. 48:126. doi: 10.1002/1531-8249(200007)48:1<126::AID-ANA21>3.0.CO;2-N, PMID: 10894228

[ref22] Carroll C. M. Li Y. M. (2016). Physiological and pathological roles of the γ-secretase complex. Brain Res. Bull. 126, 199–206. doi: 10.1016/j.brainresbull.2016.04.019, PMID: 27133790 PMC5436903

[ref23] Chan-Palay V. (1987). Somatostatin immunoreactive neurons in the human hippocampus and cortex shown by immunogold/silver intensification on vibratome sections: coexistence with neuropeptide Y neurons, and effects in Alzheimer-type dementia. J. Comp. Neurol. 260, 201–223. doi: 10.1002/cne.902600205, PMID: 2886516

[ref24] Chan-Palay V. Lang W. Haesler U. Kohler C. Yasargil G. (1986). Distribution of altered hippocampal neurons and axons immunoreactive with antisera against neuropeptide Y in Alzheimer's-type dementia. J. Comp. Neurol. 248, 376–394. doi: 10.1002/cne.902480307, PMID: 3522663

[ref25] Chartampila E. Elayouby K. S. Leary P. LaFrancois J. J. Alcantara-Gonzalez D. Jain S.. (2024). Choline supplementation in early life improves and low levels of choline can impair outcomes in a mouse model of Alzheimer's disease. eLife 12:e89884. doi: 10.7554/eLife.89889.4 PMC11192536 38904658

[ref26] Cirrito J. R. Yamada K. A. Finn M. B. Sloviter R. S. Bales K. R. May P. C.. (2005). Synaptic activity regulates interstitial fluid amyloid-β levels in vivo. Neuron 48, 913–922. doi: 10.1016/j.neuron.2005.10.028, PMID: 16364896

[ref27] Coulson E. J. May L. M. Osborne S. L. Reid K. Underwood C. K. Meunier F. A.. (2008). P75 neurotrophin receptor mediates neuronal cell death by activating GIRK channels through phosphatidylinositol 4,5-bisphosphate. J. Neurosci. 28, 315–324. doi: 10.1523/JNEUROSCI.2699-07.2008, PMID: 18171948 PMC6671158

[ref28] Coulson E. J. May L. M. Sykes A. M. Hamlin A. S. (2009). The role of the p75 neurotrophin receptor in cholinergic dysfunction in Alzheimer's disease. Neuroscientist 15, 317–323. doi: 10.1177/1073858408331376, PMID: 19458382

[ref29] Criscuolo C. Chartampila E. Ginsberg S. D. Scharfman H. E. (2024). Dentate gyrus granule cells show stability of BDNF protein expression in mossy fiber axons with age, and resistance to Alzheimer’s disease neuropathology in a mouse model. eNeuro. 11:ENEURO.0192-23.2023. doi: 10.1523/ENEURO.0192-23.2023, PMID: 38164567 PMC10913042

[ref30] Cuddy L. K. Seah C. Pasternak S. H. Rylett R. J. (2015). Differential regulation of the high-affinity choline transporter by wild-type and swedish mutant amyloid precursor protein. J. Neurochem. 134, 769–782. doi: 10.1111/jnc.13167, PMID: 25970623

[ref31] D'Alessandro M. C. B. Kanaan S. Geller M. Pratico D. Daher J. P. L. (2025). Mitochondrial dysfunction in Alzheimer's disease. Ageing Res. Rev. 107:102713. doi: 10.1016/j.arr.2025.102713, PMID: 40023293

[ref32] Davies P. (1999). Challenging the cholinergic hypothesis in Alzheimer disease. JAMA 281, 1433–1434. doi: 10.1001/jama.281.15.1433, PMID: 10217061

[ref33] Davies P. Maloney A. J. (1976). Selective loss of central cholinergic neurons in Alzheimer's disease. Lancet 2:1403. doi: 10.1016/s0140-6736(76)91936-x, PMID: 63862

[ref34] Davisson M. T. Schmidt C. Akeson E. C. (1990). Segmental trisomy of murine chromosome 16: a new model system for studying down syndrome. Prog. Clin. Biol. Res. 360, 263–280, PMID: 2147289

[ref9002] DeKosky S. T. Ikonomovic M. D. Styren S. D. Beckett L. Wisniewski S. Bennett D. A.. (2002). Upregulation of choline acetyltransferase activity in hippocampus and frontal cortex of elderly subjects with mild cognitive impairment. Ann Neurol. 51:145–55. doi: 10.1002/ana.10069 11835370

[ref35] Deller T. Katona I. Cozzari C. Frotscher M. Freund T. F. (1999). Cholinergic innervation of mossy cells in the rat fascia dentata. Hippocampus 9, 314–320. doi: 10.1002/(SICI)1098-1063(1999)9:3<314::AID-HIPO10>3.0.CO;2-7, PMID: 10401645

[ref36] Derbyshire E. Obeid R. (2020). Choline, neurological development and brain function: a systematic review focusing on the first 1000 days. Nutrients 12:1731. doi: 10.3390/nu12061731, PMID: 32531929 PMC7352907

[ref37] Duffy A. M. Morales-Corraliza J. Bermudez-Hernandez K. M. Schaner M. J. Magagna-Poveda A. Mathews P. M.. (2015). Entorhinal cortical defects in Tg2576 mice are present as early as 2-4 months of age. Neurobiol. Aging 36, 134–148. doi: 10.1016/j.neurobiolaging.2014.07.001, PMID: 25109765 PMC4268389

[ref38] Duffy A. M. Schaner M. J. Wu S. H. Staniszewski A. Kumar A. Arevalo J. C.. (2011). A selective role for ARMS/kidins220 scaffold protein in spatial memory and trophic support of entorhinal and frontal cortical neurons. Exp. Neurol. 229, 409–420. doi: 10.1016/j.expneurol.2011.03.008, PMID: 21419124 PMC3100364

[ref39] Duncan S. Brodie M. J. (2011). Sudden unexpected death in epilepsy. Epilepsy Behav. 21, 344–351. doi: 10.1016/j.yebeh.2011.04.056, PMID: 21665551

[ref40] Ezquerra M. Carnero C. Blesa R. Gelpi J. L. Ballesta F. Oliva R. (1999). A presenilin 1 mutation (ser169pro) associated with early-onset AD and myoclonic seizures. Neurology 52, 566–570. doi: 10.1212/WNL.52.3.566, PMID: 10025789

[ref41] Farrell C. Mumford P. Wiseman F. K. (2022). Rodent modeling of Alzheimer's disease in Down syndrome: in vivo and ex vivo approaches. Front. Neurosci. 16:909669. doi: 10.3389/fnins.2022.909669, PMID: 35747206 PMC9209729

[ref42] Festing MFW (1998). Inbred strains of mice: Sjl. Available online at: https://www.informatics.jax.org/inbred_strains/mouse/docs/SJL.shtml (Accessed March 2, 2025).

[ref43] Freund T. F. Antal M. (1988). GABA-containing neurons in the septum control inhibitory interneurons in the hippocampus. Nature 336, 170–173. doi: 10.1038/336170a0, PMID: 3185735

[ref44] Frotscher M. Leranth C. (1985). Cholinergic innervation of the rat hippocampus as revealed by choline acetyltransferase immunocytochemistry: a combined light and electron microscopic study. J. Comp. Neurol. 239, 237–246. doi: 10.1002/cne.902390210, PMID: 4044938

[ref45] Galdzicki Z. Siarey R. J. (2003). Understanding mental retardation in Down's syndrome using trisomy 16 mouse models. Genes Brain Behav. 2, 167–178. doi: 10.1034/j.1601-183X.2003.00024.x, PMID: 12931790

[ref46] Gautier M. K. Kelley C. M. Lee S. H. Alldred M. J. McDaid J. Mufson E. J.. (2023). Maternal choline supplementation protects against age-associated cholinergic and GABAergic basal forebrain neuron degeneration in the Ts65Dn mouse model of Down syndrome and Alzheimer's disease. Neurobiol. Dis. 188:106332. doi: 10.1016/j.nbd.2023.106332, PMID: 37890559 PMC10752300

[ref47] Gautier M. K. Kelley C. M. Lee S. H. Mufson E. J. Ginsberg S. D. (2024). Maternal choline supplementation rescues early endosome pathology in basal forebrain cholinergic neurons in the Ts65Dn mouse model of Down syndrome and Alzheimer's disease. Neurobiol. Aging 144, 30–42. doi: 10.1016/j.neurobiolaging.2024.09.002, PMID: 39265450 PMC11490376

[ref9004] George A. A. Vieira J. M. Xavier-Jackson C. Gee M. T. Cirrito J. R. Bimonte-Nelson H. A.. (2021). Implications of oligomeric amyloid-beta (oAβ_42_) signaling through α7β2-nicotinic acetylcholine receptors (nAChRs) on basal forebrain cholinergic neuronal intrinsic excitability and cognitive decline. J Neurosci. 41:555–575. doi: 10.1523/JNEUROSCI.0876-20.2020 33239400 PMC7821864

[ref48] Gimenez-Roldan S. Peraita P. Lopez Agreda J. M. Abad J. M. Esteban A. (1971). Myoclonus and photic-induced seizures in Alzheimer's disease. Eur. Neurol. 5, 215–224. 5126566 10.1159/000114073

[ref49] Green R. C. Mesulam M. M. (1988). Acetylcholinesterase fiber staining in the human hippocampus and parahippocampal gyrus. J. Comp. Neurol. 273, 488–499. doi: 10.1002/cne.902730405, PMID: 3209735

[ref50] Gu Z. Yakel J. L. (2022). Cholinergic regulation of hippocampal theta rhythm. Biomedicines 10:745. doi: 10.3390/biomedicines10040745, PMID: 35453495 PMC9027244

[ref51] Guest F. L. Rahmoune H. Guest P. C. (2020). Early diagnosis and targeted treatment strategy for improved therapeutic outcomes in Alzheimer's disease. Adv. Exp. Med. Biol. 1260, 175–191. doi: 10.1007/978-3-030-42667-5_8, PMID: 32304035

[ref52] Hartig W. Seeger J. Naumann T. Brauer K. Bruckner G. (1998). Selective in vivo fluorescence labelling of cholinergic neurons containing p75(NTR) in the rat basal forebrain. Brain Res. 808, 155–165. doi: 10.1016/S0006-8993(98)00792-6, PMID: 9767155

[ref53] Hauser W. A. (1978). Epidemiology of epilepsy. Adv. Neurol. 19, 313–339, PMID: 369325

[ref54] Hauser W. A. Kurland L. T. (1975). The epidemiology of epilepsy in Rochester, Minnesota, 1935 through 1967. Epilepsia 16, 1–66. doi: 10.1111/j.1528-1157.1975.tb04721.x, PMID: 804401

[ref55] Hauser W. A. Morris M. L. Heston L. L. Anderson V. E. (1986). Seizures and myoclonus in patients with Alzheimer's disease. Neurology 36, 1226–1230. doi: 10.1212/WNL.36.9.1226, PMID: 3092131

[ref56] Head E. Powell D. Gold B. T. Schmitt F. A. (2012). Alzheimer's disease in down syndrome. Eur. J. Neurodegener. Dis. 1, 353–364, PMID: 25285303 PMC4184282

[ref57] Hefter D. Ludewig S. Draguhn A. Korte M. (2020). Amyloid, APP, and electrical activity of the brain. Neuroscientist 26, 231–251. doi: 10.1177/1073858419882619, PMID: 31779518 PMC7222965

[ref58] Herault Y. Delabar J. M. Fisher E. M. C. Tybulewicz V. L. J. Yu E. Brault V. (2017). Rodent models in Down syndrome research: impact and future opportunities. Dis. Model. Mech. 10, 1165–1186. doi: 10.1242/dmm.029728, PMID: 28993310 PMC5665454

[ref59] Herrera D. G. Robertson H. A. (1996). Activation of c-fos in the brain. Prog. Neurobiol. 50, 83–107. doi: 10.1016/S0301-0082(96)00021-4, PMID: 8971979

[ref60] Hofmann M. E. Frazier C. J. (2010). Muscarinic receptor activation modulates the excitability of hilar mossy cells through the induction of an afterdepolarization. Brain Res. 1318, 42–51. doi: 10.1016/j.brainres.2010.01.011, PMID: 20079344 PMC2850114

[ref61] Hole K. L. Zhu B. Huggon L. Brown J. T. Mason J. M. Williams R. J. (2024). Tau(P301L) disengages from the proteosome core complex and neurogranin coincident with enhanced neuronal network excitability. Cell Death Dis. 15:429. doi: 10.1038/s41419-024-06815-2, PMID: 38890273 PMC11189525

[ref62] Hsiao K. Chapman P. Nilsen S. Eckman C. Harigaya Y. Younkin S.. (1996). Correlative memory deficits, Aβ elevation, and amyloid plaques in transgenic mice. Science 274, 99–102. doi: 10.1126/science.274.5284.99, PMID: 8810256

[ref63] Huang Y. Lemke G. (2022). Early death in a mouse model of Alzheimer's disease exacerbated by microglial loss of TAM receptor signaling. Proc. Natl. Acad. Sci. USA 119:e2204306119. doi: 10.1073/pnas.2204306119, PMID: 36191221 PMC9564325

[ref64] Hunter J. M. Cirrito J. R. Restivo J. L. Kinley R. D. Sullivan P. M. Holtzman D. M.. (2012). Emergence of a seizure phenotype in aged apolipoprotein ε4 targeted replacement mice. Brain Res. 1467, 120–132. doi: 10.1016/j.brainres.2012.05.048, PMID: 22682924

[ref65] IOM (1998). Dietary reference intakes for thiamin, riboflavin, niacin, vitamin B(6), folate, vitamin B(12), pantothenic acid, biotin, and choline. Washington (DC): National Academies Press. 23193625

[ref66] Irizarry M. C. McNamara M. Fedorchak K. Hsiao K. Hyman B. T. (1997). APPSw transgenic mice develop age-related Aβ deposits and neuropil abnormalities, but no neuronal loss in CA1. J. Neuropathol. Exp. Neurol. 56, 965–973. doi: 10.1097/00005072-199709000-00002, PMID: 9291938

[ref67] Jacobsen J. S. Wu C. C. Redwine J. M. Comery T. A. Arias R. Bowlby M.. (2006). Early-onset behavioral and synaptic deficits in a mouse model of Alzheimer's disease. Proc. Natl. Acad. Sci. USA 103, 5161–5166. doi: 10.1073/pnas.0600948103, PMID: 16549764 PMC1405622

[ref68] Jagielska A. Salaciak K. Pytka K. (2025). Beyond the blur: scopolamine's utility and limits in modeling cognitive disorders across sexes - narrative review. Ageing Res. Rev. 104:102635. doi: 10.1016/j.arr.2024.102635, PMID: 39653154

[ref9003] Jhamandas J. H. Cho C. Jassar B. Harris K. MacTavish D. Easaw J. (2001). Cellular mechanisms for amyloid beta-protein activation of rat cholinergic basal forebrain neurons. J Neurophysiol. 86:1312–20. doi: 10.1152/jn.2001.86.3.1312 11535679

[ref69] Johnson E. C. B. Ho K. Yu G. Q. Das M. Sanchez P. E. Djukic B.. (2020). Behavioral and neural network abnormalities in human APP transgenic mice resemble those of APP knock-in mice and are modulated by familial Alzheimer's disease mutations but not by inhibition of BACE1. Mol. Neurodegener. 15:53. doi: 10.1186/s13024-020-00393-5, PMID: 32921309 PMC7489007

[ref70] Judd J. M. Jasbi P. Winslow W. Serrano G. E. Beach T. G. Klein-Seetharaman J.. (2023). Inflammation and the pathological progression of Alzheimer's disease are associated with low circulating choline levels. Acta Neuropathol. 146, 565–583. doi: 10.1007/s00401-023-02616-7, PMID: 37548694 PMC10499952

[ref71] Kam K. Duffy A. M. Moretto J. LaFrancois J. J. Scharfman H. E. (2016). Interictal spikes during sleep are an early defect in the Tg2576 mouse model of β-amyloid neuropathology. Sci. Rep. 6:20119. doi: 10.1038/srep20119, PMID: 26818394 PMC4730189

[ref72] Kamenetz F. Tomita T. Hsieh H. Seabrook G. Borchelt D. Iwatsubo T.. (2003). APP processing and synaptic function. Neuron 37, 925–937. doi: 10.1016/S0896-6273(03)00124-7, PMID: 12670422

[ref9005] Kar S. Slowikowski S. P. Westaway D. Mount H. T. (2004). Interactions between beta-amyloid and central cholinergic neurons: implications for Alzheimer’s disease. J Psychiatry Neurosci. 29:427–41. 15644984 PMC524960

[ref73] Kelley C. M. Ash J. A. Powers B. E. Velazquez R. Alldred M. J. Ikonomovic M. D.. (2016). Effects of maternal choline supplementation on the septohippocampal cholinergic system in the Ts65Dn mouse model of Down syndrome. Curr. Alzheimer Res. 13, 84–96. doi: 10.2174/1567205012666150921100515, PMID: 26391045 PMC4733527

[ref74] Kennedy A. M. Newman S. McCaddon A. Ball J. Roques P. Mullan M.. (1993). Familial Alzheimer's disease. A pedigree with a mis-sense mutation in the amyloid precursor protein gene (amyloid precursor protein 717 valine-->glycine). Brain 116, 309–324. doi: 10.1093/brain/116.2.309, PMID: 8461968

[ref75] Kleen J. K. Scott R. C. Holmes G. L. Lenck-Santini P. P. (2010). Hippocampal interictal spikes disrupt cognition in rats. Ann. Neurol. 67, 250–257. doi: 10.1002/ana.21896, PMID: 20225290 PMC2926932

[ref76] Kleen J. K. Scott R. C. Holmes G. L. Roberts D. W. Rundle M. M. Testorf M.. (2013). Hippocampal interictal epileptiform activity disrupts cognition in humans. Neurology 81, 18–24. doi: 10.1212/WNL.0b013e318297ee50, PMID: 23685931 PMC3770206

[ref77] Kleen J. K. Scott R. C. Lenck-Santini P. P. Holmes G. L. (2012). “Cognitive and behavioral co-morbidities of epilepsy” in Jasper's basic mechanisms of the epilepsies. eds. Avoli M. Rogawski M. A. Olsen R. W. Delgado-Escueta A. V.. 4th ed (Bethesda (MD): Noebels JL). 22787667

[ref78] Konings S. C. Torres-Garcia L. Martinsson I. Gouras G. K. (2021). Astrocytic and neuronal apolipoprotein e isoforms differentially affect neuronal excitability. Front. Neurosci. 15:734001. doi: 10.3389/fnins.2021.734001, PMID: 34621153 PMC8490647

[ref79] Kumar A. Lana E. Kumar R. Lithner C. U. Darreh-Shori T. (2018). Soluble Aβ42 acts as allosteric activator of the core cholinergic enzyme choline acetyltransferase. Front. Mol. Neurosci. 11:327. doi: 10.3389/fnmol.2018.00327, PMID: 30271321 PMC6146036

[ref80] Lai N. Li Z. Xu C. Wang Y. Chen Z. (2023). Diverse nature of interictal oscillations: EEG-based biomarkers in epilepsy. Neurobiol. Dis. 177:105999. doi: 10.1016/j.nbd.2023.105999, PMID: 36638892

[ref81] Lallement G. Dorandeu F. Filliat P. Carpentier P. Baille V. Blanchet G. (1998). Medical management of organophosphate-induced seizures. J. Physiol. Paris 92, 369–373. doi: 10.1016/S0928-4257(99)80007-2, PMID: 9789839

[ref82] Lam A. D. Deck G. Goldman A. Eskandar E. N. Noebels J. Cole A. J. (2017). Silent hippocampal seizures and spikes identified by foramen ovale electrodes in Alzheimer's disease. Nat. Med. 23, 678–680. doi: 10.1038/nm.4330, PMID: 28459436 PMC5461182

[ref83] Lam A. D. Sarkis R. A. Pellerin K. R. Jing J. Dworetzky B. A. Hoch D. B.. (2020). Association of epileptiform abnormalities and seizures in Alzheimer disease. Neurology 95, e2259–e2270. doi: 10.1212/WNL.0000000000010612, PMID: 32764101 PMC7713786

[ref84] Lamoureux L. Marottoli F. M. Tseng K. Y. Tai L. M. (2021). Apoe4 promotes tonic-clonic seizures, an effect modified by familial Alzheimer's disease mutations. Front. Cell Dev. Biol. 9:656521. doi: 10.3389/fcell.2021.656521, PMID: 33796539 PMC8007905

[ref85] Lara S. Laureani Á. Aranda G. Toledo R. García L. Coria G.. (2022). Current opinion on the use of c-Fos in neuroscience. NeuroSci 3, 687–702. doi: 10.3390/neurosci3040050, PMID: 39483772 PMC11523728

[ref86] Leranth C. Hajszan T. (2007). Extrinsic afferent systems to the dentate gyrus. Prog. Brain Res. 163, 63–84. doi: 10.1016/S0079-6123(07)63004-0, PMID: 17765712 PMC1989689

[ref87] Levesque M. Ragsdale D. Avoli M. (2019). Evolving mechanistic concepts of epileptiform synchronization and their relevance in curing focal epileptic disorders. Curr. Neuropharmacol. 17, 830–842. doi: 10.2174/1570159X17666181127124803, PMID: 30479217 PMC7052840

[ref88] Levesque M. Wang S. Macey-Dare A. D. B. Salami P. Avoli M. (2023). Evolution of interictal activity in models of mesial temporal lobe epilepsy. Neurobiol. Dis. 180:106065. doi: 10.1016/j.nbd.2023.106065, PMID: 36907521

[ref89] Lind D. Franken S. Kappler J. Jankowski J. Schilling K. (2005). Characterization of the neuronal marker NeuN as a multiply phosphorylated antigen with discrete subcellular localization. J. Neurosci. Res. 79, 295–302. doi: 10.1002/jnr.20354, PMID: 15605376

[ref90] Lisgaras C. P. Scharfman H. E. (2023a). Interictal spikes in Alzheimer's disease: preclinical evidence for dominance of the dentate gyrus and cholinergic control by the medial septum. Neurobiol. Dis. 187:106294. doi: 10.1016/j.nbd.2023.106294, PMID: 37714307 PMC10617404

[ref91] Lisgaras C. P. Scharfman H. E. (2023b). High-frequency oscillations (250-500 Hz) in animal models of Alzheimer's disease and two animal models of epilepsy. Epilepsia 64, 231–246. doi: 10.1111/epi.17462, PMID: 36346209 PMC10501735

[ref92] Liu S. Shen Y. Shultz S. R. Nguyen A. Hovens C. Adlard P. A.. (2017). Accelerated kindling epileptogenesis in Tg4510 tau transgenic mice, but not in tau knockout mice. Epilepsia 58, e136–e141. doi: 10.1111/epi.13847, PMID: 28710841

[ref93] Liu T. Wang Y. Wang J. Ren C. Chen H. Zhang J. (2022). Dyrk1a inhibitors for disease therapy: current status and perspectives. Eur. J. Med. Chem. 229:114062. doi: 10.1016/j.ejmech.2021.114062, PMID: 34954592

[ref94] Loreth D. Ozmen L. Revel F. G. Knoflach F. Wetzel P. Frotscher M.. (2012). Selective degeneration of septal and hippocampal GABAergic neurons in a mouse model of amyloidosis and tauopathy. Neurobiol. Dis. 47, 1–12. doi: 10.1016/j.nbd.2012.03.011, PMID: 22426397

[ref95] Luebke J. I. Weaver C. M. Rocher A. B. Rodriguez A. Crimins J. L. Dickstein D. L.. (2010). Dendritic vulnerability in neurodegenerative disease: insights from analyses of cortical pyramidal neurons in transgenic mouse models. Brain Struct. Funct. 214, 181–199. doi: 10.1007/s00429-010-0244-2, PMID: 20177698 PMC3045830

[ref96] Mangone C. A. Castano E. M. Levy E. Abiusi G. Wisniewski T. Marques M. R.. (1995). Early onset Alzheimer's disease in a south american pedigree from Argentina. Acta Neurol. Scand. 91, 6–13. doi: 10.1111/j.1600-0404.1995.tb05835.x, PMID: 7732777

[ref97] Marksteiner J. Sperk G. Maas D. (1989). Differential increases in brain levels of neuropeptide Y and vasoactive intestinal polypeptide after kainic acid-induced seizures in the rat. Naunyn Schmiedeberg's Arch. Pharmacol. 339, 173–177. doi: 10.1007/BF00165140, PMID: 2566924

[ref98] Martinez J. L. Zammit M. D. West N. R. Christian B. T. Bhattacharyya A. (2021). Basal forebrain cholinergic neurons: linking down syndrome and Alzheimer's disease. Front. Aging Neurosci. 13:703876. doi: 10.3389/fnagi.2021.703876, PMID: 34322015 PMC8311593

[ref99] Martinez-Losa M. Tracy T. E. Ma K. Verret L. Clemente-Perez A. Khan A. S.. (2018). Nav1.1-overexpressing interneuron transplants restore brain rhythms and cognition in a mouse model of Alzheimer's disease. Neuron 98, 75–89 e75. doi: 10.1016/j.neuron.2018.02.029, PMID: 29551491 PMC5886814

[ref100] Mattson M. P. (1997). Cellular actions of β-amyloid precursor protein and its soluble and fibrillogenic derivatives. Physiol. Rev. 77, 1081–1132. doi: 10.1152/physrev.1997.77.4.1081, PMID: 9354812

[ref101] McClung C. A. Ulery P. G. Perrotti L. I. Zachariou V. Berton O. Nestler E. J. (2004). ΔFosb: a molecular switch for long-term adaptation in the brain. Mol. Brain Res. 132, 146–154. doi: 10.1016/j.molbrainres.2004.05.014, PMID: 15582154

[ref102] Meckler X. Checler F. (2016). Presenilin 1 and presenilin 2 target γ-secretase complexes to distinct cellular compartments. J. Biol. Chem. 291, 12821–12837. doi: 10.1074/jbc.M115.708297, PMID: 27059953 PMC4933450

[ref103] Mellott T. J. Huleatt O. M. Shade B. N. Pender S. M. Liu Y. B. Slack B. E.. (2017). Perinatal choline supplementation reduces amyloidosis and increases choline acetyltransferase expression in the hippocampus of the APPSwePS1de9 Alzheimer's disease model mice. PLoS One 12:e0170450. doi: 10.1371/journal.pone.0170450, PMID: 28103298 PMC5245895

[ref104] Mendez M. Lim G. (2003). Seizures in elderly patients with dementia: epidemiology and management. Drugs Aging 20, 791–803. doi: 10.2165/00002512-200320110-00001, PMID: 12964886

[ref105] Minkeviciene R. Rheims S. Dobszay M. B. Zilberter M. Hartikainen J. Fulop L.. (2009). Amyloid β-induced neuronal hyperexcitability triggers progressive epilepsy. J. Neurosci. 29, 3453–3462. doi: 10.1523/JNEUROSCI.5215-08.2009, PMID: 19295151 PMC6665248

[ref106] Mizuseki K. Miyawaki H. (2023). Fast network oscillations during non-REM sleep support memory consolidation. Neurosci. Res. 189, 3–12. doi: 10.1016/j.neures.2022.12.019, PMID: 36581177

[ref107] Moon J. Chen M. Gandhy S. U. Strawderman M. Levitsky D. A. Maclean K. N.. (2010). Perinatal choline supplementation improves cognitive functioning and emotion regulation in the Ts65Dn mouse model of Down syndrome. Behav. Neurosci. 124, 346–361. doi: 10.1037/a0019590, PMID: 20528079 PMC2955960

[ref108] Mucke L. Masliah E. Yu G. Q. Mallory M. Rockenstein E. M. Tatsuno G.. (2000). High-level neuronal expression of Aβ 1-42 in wild-type human amyloid protein precursor transgenic mice: Synaptotoxicity without plaque formation. J. Neurosci. 20, 4050–4058. doi: 10.1523/JNEUROSCI.20-11-04050.2000, PMID: 10818140 PMC6772621

[ref109] Mufson E. J. Ginsberg S. D. Ikonomovic M. D. DeKosky S. T. (2003). Human cholinergic basal forebrain: Chemoanatomy and neurologic dysfunction. J. Chem. Neuroanat. 26, 233–242. doi: 10.1016/S0891-0618(03)00068-1, PMID: 14729126

[ref110] Mufson E. J. Ikonomovic M. D. Counts S. E. Perez S. E. Malek-Ahmadi M. Scheff S. W.. (2016). Molecular and cellular pathophysiology of preclinical Alzheimer's disease. Behav. Brain Res. 311, 54–69. doi: 10.1016/j.bbr.2016.05.030, PMID: 27185734 PMC4931948

[ref111] Nalivaeva N. N. Belyaev N. D. Zhuravin I. A. Turner A. J. (2012). The Alzheimer's amyloid-degrading peptidase, neprilysin: can we control it? Int. J. Alzheimers Dis. 2012:383796. doi: 10.1155/2012/383796, PMID: 22900228 PMC3412116

[ref112] Nedergaard M. Goldman S. A. (2020). Glymphatic failure as a final common pathway to dementia. Science 370, 50–56. doi: 10.1126/science.abb8739, PMID: 33004510 PMC8186542

[ref113] Nilsson P. Saito T. Saido T. C. (2014). New mouse model of Alzheimer's. ACS Chem. Neurosci. 5, 499–502. doi: 10.1021/cn500105p, PMID: 24852598 PMC4102956

[ref114] Nixon R. A. Rubinsztein D. C. (2024). Mechanisms of autophagy-lysosome dysfunction in neurodegenerative diseases. Nat. Rev. Mol. Cell Biol. 25, 926–946. doi: 10.1038/s41580-024-00757-5, PMID: 39107446 PMC12239022

[ref115] Opeskin K. (1996). Cerebral amyloid angiopathy. A review. Am J Forensic Med Pathol 17, 248–254. doi: 10.1097/00000433-199609000-00013, PMID: 8870877

[ref116] Paesler K. Xie K. Hettich M. M. Siwek M. E. Ryan D. P. Schroder S.. (2015). Limited effects of an eif2aS51a allele on neurological impairments in the 5xFAD mouse model of Alzheimer's disease. Neural Plast. 2015:825157. doi: 10.1155/2015/825157, PMID: 25883808 PMC4391319

[ref117] Palop J. J. Chin J. Roberson E. D. Wang J. Thwin M. T. Bien-Ly N.. (2007). Aberrant excitatory neuronal activity and compensatory remodeling of inhibitory hippocampal circuits in mouse models of Alzheimer's disease. Neuron 55, 697–711. doi: 10.1016/j.neuron.2007.07.025, PMID: 17785178 PMC8055171

[ref118] Palop J. J. Mucke L. (2009). Epilepsy and cognitive impairments in Alzheimer disease. Arch. Neurol. 66, 435–440. doi: 10.1001/archneurol.2009.15, PMID: 19204149 PMC2812914

[ref119] Palop J. J. Mucke L. Roberson E. D. (2011). Quantifying biomarkers of cognitive dysfunction and neuronal network hyperexcitability in mouse models of Alzheimer's disease: depletion of calcium-dependent proteins and inhibitory hippocampal remodeling. Methods Mol. Biol. 670, 245–262. doi: 10.1007/978-1-60761-744-0_17, PMID: 20967595 PMC8153735

[ref120] Parikh V. Bernard C. S. Naughton S. X. Yegla B. (2014). Interactions between Aβ oligomers and presynaptic cholinergic signaling: age-dependent effects on attentional capacities. Behav. Brain Res. 274, 30–42. doi: 10.1016/j.bbr.2014.07.046, PMID: 25101540 PMC4179990

[ref121] Pei Y. Rogan S. C. Yan F. Roth B. L. (2008). Engineered GPCRs as tools to modulate signal transduction. Physiology (Bethesda) 23, 313–321. doi: 10.1152/physiol.00025.2008, PMID: 19074739

[ref122] Piccarducci R. Giacomelli C. Bertilacchi M. S. Benito-Martinez A. Di Giorgi N. Daniele S.. (2023). Apolipoprotein E ε4 triggers neurotoxicity via cholesterol accumulation, acetylcholine dyshomeostasis, and PKCε mislocalization in cholinergic neuronal cells. Biochim. Biophys. Acta Mol. basis Dis. 1869:166793. doi: 10.1016/j.bbadis.2023.166793, PMID: 37336366

[ref123] Powers B. E. Velazquez R. Strawderman M. S. Ginsberg S. D. Mufson E. J. Strupp B. J. (2021). Maternal choline supplementation as a potential therapy for Down syndrome: assessment of effects throughout the lifespan. Front. Aging Neurosci. 13:723046. doi: 10.3389/fnagi.2021.723046, PMID: 34690739 PMC8527982

[ref124] Price D. L. Whitehouse P. J. Struble R. G. Coyle J. T. Clark A. W. Delong M. R.. (1982). Alzheimer's disease and Down's syndrome. Ann. N. Y. Acad. Sci. 396, 145–164. doi: 10.1111/j.1749-6632.1982.tb26850.x, PMID: 6217772

[ref125] Prince D. A. Avoli M. (2024). “The paroxysmal depolarizing shift: the first cellular marker of focal epileptogenesis” in Jasper's basic mechanisms of the epilepsies. eds. Avoli M. Rogawski M. A. Vezzani A. Delgado-Escueta A. V.. 5th ed (New York: Noebels JL), 5–14. 39637141

[ref126] Rainero I. Bergamini L. Bruni A. C. Ferini-Strambi L. Foncin J. F. Gei G.. (1994). A new italian pedigree with early-onset Alzheimer's disease. J. Geriatr. Psychiatry Neurol. 7, 28–32. doi: 10.1177/089198879400700106, PMID: 8192827

[ref127] Rajmohan R. Reddy P. H. (2017). Amyloid-β and phosphorylated tau accumulations cause abnormalities at synapses of Alzheimer's disease neurons. J. Alzheimers Dis. 57, 975–999. doi: 10.3233/JAD-160612, PMID: 27567878 PMC5793225

[ref128] Ramos B. Baglietto-Vargas D. del Rio J. C. Moreno-Gonzalez I. Santa-Maria C. Jimenez S.. (2006). Early neuropathology of somatostatin/npy GABAergic cells in the hippocampus of a PS1xAPP transgenic model of Alzheimer's disease. Neurobiol. Aging 27, 1658–1672. doi: 10.1016/j.neurobiolaging.2005.09.022, PMID: 16271420

[ref129] Ren Y. Zeng Y. Wu Y. Zhang Q. Xiao X. (2024). Maternal methyl donor supplementation: a potential therapy for metabolic disorder in offspring. J. Nutr. Biochem. 124:109533. doi: 10.1016/j.jnutbio.2023.109533, PMID: 37977406

[ref130] Richter M. C. Ludewig S. Winschel A. Abel T. Bold C. Salzburger L. R.. (2018). Distinct in vivo roles of secreted APP ectodomain variants appsα and APPsβ in regulation of spine density, synaptic plasticity, and cognition. EMBO J. 37:798335. doi: 10.15252/embj.201798335, PMID: 29661886 PMC5983155

[ref131] Risse S. C. Lampe T. H. Bird T. D. Nochlin D. Sumi S. M. Keenan T.. (1990). Myoclonus, seizures, and paratonia in Alzheimer disease. Alzheimer Dis. Assoc. Disord. 4, 217–225. doi: 10.1097/00002093-199040400-00003, PMID: 2264979

[ref132] Roberson E. D. Scearce-Levie K. Palop J. J. Yan F. Cheng I. H. Wu T.. (2007). Reducing endogenous tau ameliorates amyloid β-induced deficits in an Alzheimer's disease mouse model. Science 316, 750–754. doi: 10.1126/science.1141736, PMID: 17478722

[ref133] Romanelli M. F. Morris J. C. Ashkin K. Coben L. A. (1990). Advanced Alzheimer's disease is a risk factor for late-onset seizures. Arch. Neurol. 47, 847–850. doi: 10.1001/archneur.1990.00530080029006, PMID: 2375689

[ref134] Rossi A. Galla L. Gomiero C. Zentilin L. Giacca M. Giorgio V.. (2021). Calcium signaling and mitochondrial function in presenilin 2 knock-out mice: looking for any loss-of-function phenotype related to Alzheimer's disease. Cells 10:204. doi: 10.3390/cells10020204, PMID: 33494218 PMC7909802

[ref135] Rossor M. N. Emson P. C. Mountjoy C. Q. Roth M. Iversen L. L. (1980). Reduced amounts of immunoreactive somatostatin in the temporal cortex in senile dementia of Alzheimer type. Neurosci. Lett. 20, 373–377. doi: 10.1016/0304-3940(80)90177-9, PMID: 6108540

[ref136] Roth J. R. Rush T. Thompson S. J. Aldaher A. R. Dunn T. B. Mesina J. S.. (2024). Development of small-molecule tau-SH3 interaction inhibitors that prevent amyloid-β toxicity and network hyperexcitability. Neurotherapeutics 21:e00291. doi: 10.1016/j.neurot.2023.10.001, PMID: 38241154 PMC10903085

[ref137] Rouse S. T. Levey A. I. (1997). Muscarinic acetylcholine receptor immunoreactivity after hippocampal commissural/associational pathway lesions: evidence for multiple presynaptic receptor subtypes. J. Comp. Neurol. 380, 382–394. doi: 10.1002/(SICI)1096-9861(19970414)380:3<382::AID-CNE7>3.0.CO;2-Z, PMID: 9087520

[ref138] Saito T. Matsuba Y. Mihira N. Takano J. Nilsson P. Itohara S.. (2014). Single APP knock-in mouse models of Alzheimer's disease. Nat. Neurosci. 17, 661–663. doi: 10.1038/nn.3697, PMID: 24728269

[ref139] Salehi A. Ashford J. W. Mufson E. J. (2016). The link between Alzheimer's disease and Down syndrome. A historical perspective. Curr. Alzheimer Res. 13, 2–6. doi: 10.2174/1567205012999151021102914, PMID: 26487155 PMC6368451

[ref140] Sanchez M. P. Garcia-Cabrero A. M. Sanchez-Elexpuru G. Burgos D. F. Serratosa J. M. (2018). Tau-induced pathology in epilepsy and dementia: notions from patients and animal models. Int. J. Mol. Sci. 19:1092. doi: 10.3390/ijms19041092, PMID: 29621183 PMC5979593

[ref141] Scarpelli S. Bartolacci C. D'Atri A. Gorgoni M. De Gennaro L. (2019). The functional role of dreaming in emotional processes. Front. Psychol. 10:459. doi: 10.3389/fpsyg.2019.00459, PMID: 30930809 PMC6428732

[ref142] Scharfman H. E. (2016). The enigmatic mossy cell of the dentate gyrus. Nat. Rev. Neurosci. 17, 562–575. doi: 10.1038/nrn.2016.87, PMID: 27466143 PMC5369357

[ref143] Schliebs R. Arendt T. (2011). The cholinergic system in aging and neuronal degeneration. Behav. Brain Res. 221, 555–563. doi: 10.1016/j.bbr.2010.11.058, PMID: 21145918

[ref144] Shao E. Chang C. W. Li Z. Yu X. Ho K. Zhang M.. (2022). Tau ablation in excitatory neurons and postnatal tau knockdown reduce epilepsy, sudep, and autism behaviors in a Dravet syndrome model. Sci. Transl. Med. 14:eabm5527. doi: 10.1126/scitranslmed.abm5527, PMID: 35476595 PMC9102397

[ref145] Siwek M. E. Muller R. Henseler C. Trog A. Lundt A. Wormuth C.. (2015). Altered theta oscillations and aberrant cortical excitatory activity in the 5XFAD model of Alzheimer's disease. Neural Plast. 2015:781731. doi: 10.1155/2015/781731, PMID: 25922768 PMC4398951

[ref146] Socha M. W. Flis W. Wartega M. (2024). Epigenetic genome modifications during pregnancy: the impact of essential nutritional supplements on DNA methylation. Nutrients 16:678. doi: 10.3390/nu16050678, PMID: 38474806 PMC10934520

[ref147] Sperfeld A. D. Collatz M. B. Baier H. Palmbach M. Storch A. Schwarz J.. (1999). FTDP-17: an early-onset phenotype with parkinsonism and epileptic seizures caused by a novel mutation. Ann. Neurol. 46, 708–715. doi: 10.1002/1531-8249(199911)46:5<708::AID-ANA5>3.0.CO;2-K, PMID: 10553987

[ref148] Staba R. J. Bragin A. (2011). High-frequency oscillations and other electrophysiological biomarkers of epilepsy: underlying mechanisms. Biomark. Med 5, 545–556. doi: 10.2217/bmm.11.72, PMID: 22003903 PMC3233380

[ref149] Stark S. M. Yassa M. A. Lacy J. W. Stark C. E. (2013). A task to assess behavioral pattern separation (bps) in humans: data from healthy aging and mild cognitive impairment. Neuropsychologia 51, 2442–2449. doi: 10.1016/j.neuropsychologia.2012.12.014, PMID: 23313292 PMC3675184

[ref150] Strupp B. J. Powers B. E. Velazquez R. Ash J. A. Kelley C. M. Alldred M. J.. (2016). Maternal choline supplementation: a potential prenatal treatment for down syndrome and Alzheimer's disease. Curr. Alzheimer Res. 13, 97–106. doi: 10.2174/1567205012666150921100311, PMID: 26391046 PMC4733524

[ref151] Taipala E. Pfitzer J. C. Hellums M. Reed M. N. Gramlich M. W. (2022). Rtg(tau(P301L))4510 mice exhibit increased vGLUT1 in hippocampal presynaptic glutamatergic vesicles and increased extracellular glutamate release. Front. Synaptic Neurosci. 14:925546. doi: 10.3389/fnsyn.2022.925546, PMID: 35989711 PMC9383415

[ref152] Tandon A. Fraser P. (2002). The presenilins. Genome Biol. 3:reviews3014. doi: 10.1186/gb-2002-3-11-reviews3014, PMID: 12429067 PMC244923

[ref153] Todorovic M. S. Cowan M. L. Balint C. A. Sun C. Kapur J. (2012). Characterization of status epilepticus induced by two organophosphates in rats. Epilepsy Res. 101, 268–276. doi: 10.1016/j.eplepsyres.2012.04.014, PMID: 22578704 PMC3419801

[ref154] Tolia A. De Strooper B. (2009). Structure and function of γ-secretase. Semin. Cell Dev. Biol. 20, 211–218. doi: 10.1016/j.semcdb.2008.10.007, PMID: 19007897

[ref155] Turski W. A. Cavalheiro E. A. Schwarz M. Czuczwar S. J. Kleinrok Z. Turski L. (1983a). Limbic seizures produced by pilocarpine in rats: Behavioural, electroencephalographic and neuropathological study. Behav. Brain Res. 9, 315–335. doi: 10.1016/0166-4328(83)90136-5, PMID: 6639740

[ref156] Turski W. A. Cavalheiro E. A. Turski L. Kleinrok Z. (1983b). Intrahippocampal bethanechol in rats: Behavioural, electroencephalographic and neuropathological correlates. Behav. Brain Res. 7, 361–370. doi: 10.1016/0166-4328(83)90026-8, PMID: 6132610

[ref157] Unal-Cevik I. Kilinc M. Gursoy-Ozdemir Y. Gurer G. Dalkara T. (2004). Loss of NeuN immunoreactivity after cerebral ischemia does not indicate neuronal cell loss: a cautionary note. Brain Res. 1015, 169–174. doi: 10.1016/j.brainres.2004.04.032, PMID: 15223381

[ref158] Vande Vyver M. Barker-Haliski M. Aourz N. Nagels G. Bjerke M. Engelborghs S.. (2022). Higher susceptibility to 6 Hz corneal kindling and lower responsiveness to antiseizure drugs in mouse models of Alzheimer's disease. Epilepsia 63, 2703–2715. doi: 10.1111/epi.17355, PMID: 35775150 PMC9804582

[ref159] Velazquez R. Ferreira E. Winslow W. Dave N. Piras I. S. Naymik M.. (2020). Maternal choline supplementation ameliorates Alzheimer's disease pathology by reducing brain homocysteine levels across multiple generations. Mol. Psychiatry 25, 2620–2629. doi: 10.1038/s41380-018-0322-z, PMID: 30622336 PMC6697226

[ref160] Verret L. Mann E. O. Hang G. B. Barth A. M. Cobos I. Ho K.. (2012). Inhibitory interneuron deficit links altered network activity and cognitive dysfunction in Alzheimer model. Cell 149, 708–721. doi: 10.1016/j.cell.2012.02.046, PMID: 22541439 PMC3375906

[ref161] Volicer L. Smith S. Volicer B. J. (1995). Effect of seizures on progression of dementia of the Alzheimer type. Dementia 6, 258–263. doi: 10.1159/000106956, PMID: 8528372

[ref162] Vossel K. A. Beagle A. J. Rabinovici G. D. Shu H. Lee S. E. Naasan G.. (2013). Seizures and epileptiform activity in the early stages of Alzheimer disease. JAMA Neurol. 70, 1158–1166. doi: 10.1001/jamaneurol.2013.136, PMID: 23835471 PMC4013391

[ref164] Vossel K. A. Ranasinghe K. G. Beagle A. J. Mizuiri D. Honma S. M. Dowling A. F.. (2016). Incidence and impact of subclinical epileptiform activity in Alzheimer's disease. Ann. Neurol. 80, 858–870. doi: 10.1002/ana.24794, PMID: 27696483 PMC5177487

[ref163] Vossel K. Ranasinghe K. G. Beagle A. J. La A. Ah Pook K. Castro M.. (2021). Effect of levetiracetam on cognition in patients with Alzheimer disease with and without epileptiform activity: a randomized clinical trial. JAMA Neurol. 78, 1345–1354. doi: 10.1001/jamaneurol.2021.3310, PMID: 34570177 PMC8477304

[ref165] Watson C. J. Baghdoyan H. A. Lydic R. (2010). Neuropharmacology of sleep and wakefulness. Sleep Med. Clin. 5, 513–528. doi: 10.1016/j.jsmc.2010.08.003, PMID: 21278831 PMC3026477

[ref166] Wilcox K. S. Vezzani A. (2014). Does brain inflammation mediate pathological outcomes in epilepsy? Adv. Exp. Med. Biol. 813, 169–183. doi: 10.1007/978-94-017-8914-1_14, PMID: 25012376 PMC4867105

[ref167] Wolfe M. S. (2009). γ-secretase in biology and medicine. Semin. Cell Dev. Biol. 20, 219–224. doi: 10.1016/j.semcdb.2008.12.011, PMID: 19162210

[ref168] Wolfe M. S. (2025). Presenilin, γ-secretase, and the search for pathogenic triggers of Alzheimer's disease. Biochemistry 64, 1662–1672. doi: 10.1021/acs.biochem.4c00830, PMID: 39996369 PMC13137448

[ref169] Xia M. Cheng X. Yi R. Gao D. Xiong J. (2016). The binding receptors of Aβ: an alternative therapeutic target for Alzheimer's disease. Mol. Neurobiol. 53, 455–471. doi: 10.1007/s12035-014-8994-0, PMID: 25465238

[ref170] Xolalpa-Cueva L. Garcia-Carlos C. A. Villasenor-Zepeda R. Orta-Salazar E. Diaz-Cintra S. Pena-Ortega F.. (2022). Hyperphosphorylated tau relates to improved cognitive performance and reduced hippocampal excitability in the young rTg4510 mouse model of tauopathy. J. Alzheimers Dis. 87, 529–543. doi: 10.3233/JAD-215186, PMID: 35342085

[ref171] Zeisel S. H. da Costa K. A. (2009). Choline: an essential nutrient for public health. Nutr. Rev. 67, 615–623. doi: 10.1111/j.1753-4887.2009.00246.x, PMID: 19906248 PMC2782876

[ref172] Zhou J. Benoit M. Sharoar M. G. (2022). Recent advances in pre-clinical diagnosis of Alzheimer's disease. Metab. Brain Dis. 37, 1703–1725. doi: 10.1007/s11011-021-00733-4, PMID: 33900524

